# Genome sequence of the insect pathogenic fungus *Cordyceps militaris*, a valued traditional chinese medicine

**DOI:** 10.1186/gb-2011-12-11-r116

**Published:** 2011-11-23

**Authors:** Peng Zheng, Yongliang Xia, Guohua Xiao, Chenghui Xiong, Xiao Hu, Siwei Zhang, Huajun Zheng, Yin Huang, Yan Zhou, Shengyue Wang, Guo-Ping Zhao, Xingzhong Liu, Raymond J St Leger, Chengshu Wang

**Affiliations:** 1 Key Laboratory of Insect Developmental and Evolutionary Biology, Institute of Plant Physiology and Ecology, Shanghai Institutes for Biological Sciences, Chinese Academy of Sciences, 300 Fenglin Road, Shanghai 200032, China; 2 Chinese National Human Genome Center at Shanghai, 250 Bibo Road, Shanghai 201203, China; 3 Institute of Microbiology, Chinese Academy of Sciences, 1 West Beichen Road, Beijing 100101, China; 4 Department of Entomology, University of Maryland, 4112 Plant Sciences Building, College Park, Maryland 20742, USA

## Abstract

**Background:**

Species in the ascomycete fungal genus *Cordyceps *have been proposed to be the teleomorphs of *Metarhizium *species. The latter have been widely used as insect biocontrol agents. *Cordyceps *species are highly prized for use in traditional Chinese medicines, but the genes responsible for biosynthesis of bioactive components, insect pathogenicity and the control of sexuality and fruiting have not been determined.

**Results:**

Here, we report the genome sequence of the type species *Cordyceps militaris*. Phylogenomic analysis suggests that different species in the *Cordyceps*/*Metarhizium *genera have evolved into insect pathogens independently of each other, and that their similar large secretomes and gene family expansions are due to convergent evolution. However, relative to other fungi, including *Metarhizium *spp., many protein families are reduced in *C. militaris*, which suggests a more restricted ecology. Consistent with its long track record of safe usage as a medicine, the *Cordyceps *genome does not contain genes for known human mycotoxins. We establish that *C. militaris *is sexually heterothallic but, very unusually, fruiting can occur without an opposite mating-type partner. Transcriptional profiling indicates that fruiting involves induction of the Zn2Cys6-type transcription factors and MAPK pathway; unlike other fungi, however, the PKA pathway is not activated.

**Conclusions:**

The data offer a better understanding of *Cordyceps *biology and will facilitate the exploitation of medicinal compounds produced by the fungus.

## Background

The Ascomycete genus *Cordyceps *includes over 500 species that are pathogens of arthropods. *Cordyceps militaris *(CCM) is the type species and occurs throughout much of the Northern Hemisphere as a pathogen of lepidopteran insect pupae [1]. *C. militaris *is readily characterized by the sexual fruiting bodies forming on mycosed pupae, the structures giving the fungus its common name of 'pupa grass' in China. Anamorphic *Cordyceps *species, such as *Beauveria *spp., *Metarhizium *spp. and *Paecilomyces *spp., have been developed as insect biocontrol agents [2,3]. Although *C. militaris *and *Cordyceps sinensis *(syn. *Ophiocordyceps sinensis*) are best known as traditional Chinese medicines, they are also increasingly being studied and used in the West [4,5]. An array of pharmacologically active components has been identified, including cordycepin, cordycepic acids, polysaccharides and macrolides [6]. Cordycepin (3'-deoxyadenosine) has so far only been reported in *C. militaris *and is a broad spectrum antimicrobial [5] and polyadenylation inhibitor that is currently undergoing clinical trials against cancers [7]. The biosynthetic pathway of cordycepin production has not been determined.

**Figure 1:**
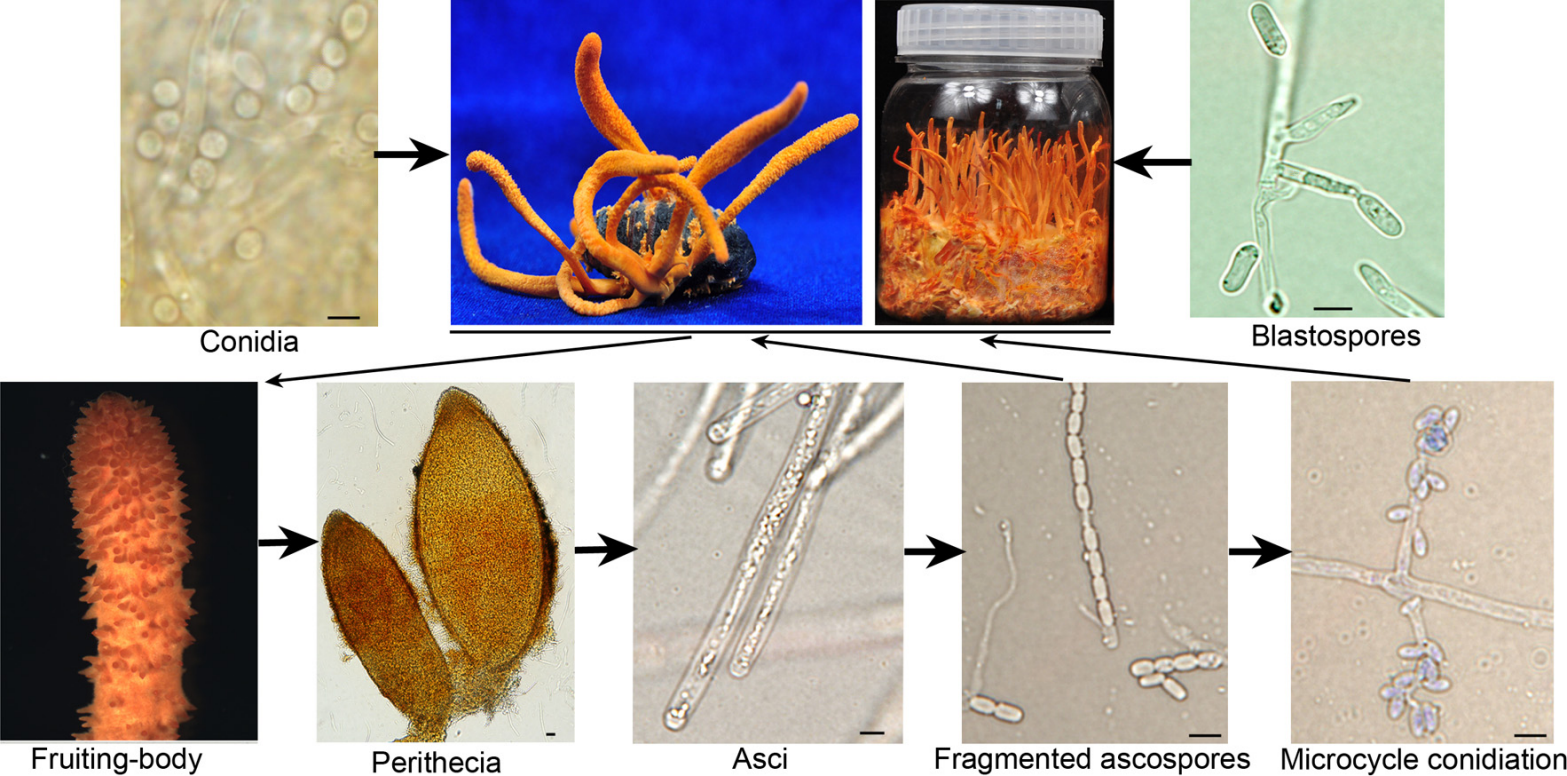
**Life cycle and phenotypic polymorphism of *C. militaris***. The round conidia (from a solid culture) or the bar shaped blastospores (from a liquid culture) were inoculated onto caterpillar pupa or rice medium and incubated for up to 60 days. The resulting fertile fruiting bodies have protruded perithecia that contain asci. The ejected linear ascospores fragment and germinate to produce secondary pear-shaped conidia under nutrient poor conditions, that is, micro-cycle conidiation. Both the ascospores and secondary conidia can infect caterpillars. Scale bar: 5 μl.

In spite of their market values - for example, > $10,000 per kilo for the fruiting bodies of the un-cultivatable *C. sinensis *[8] - very little is known about sex and developmental processes in *Cordyceps *species, and remedying this deficiency should help in production/cultivation of these enigmatic fungi. *C. militaris *is notable as it readily performs sexual reproduction on artificial media and is thus a good target for studying the molecular underpinnings of sex and development in *Cordyceps *spp. (Figure 1). *C. militaris *also has the potential to be a versatile new model for studying the evolution of sex and reproductive structures. While current fungal models have provided numerous insights into the evolution of sex [9], there is still much to be understood about the mechanisms, evolution and ecological impact of sexuality in fungi. This is, in part, because fungal mating and sexual cycles are often complicated; for example, aspergilli have both self-fertile (homothallism) and self-sterile (heterothallism) mating systems [10].

There is also much to be learnt about the nature and evolution of interactions of *Cordyceps *spp. with their hosts and with the wider environment. As entomopathogenicity appears to have evolved independently in *Cordyceps *and two *Metarhizium *species [11], comparative genomics will provide independent assessments of what is required to be entomopathogenic, identify the degree to which evolution between these fungi has been convergent, and identify the genomic basis of their differing physiologies and host-specificity. Last but not least, genomic sequencing of *C. militaris *will enable a systematic exploration of the biology and pharmaceuticals underlying the widespread medical impact of *Cordyceps *spp., and identify potential safety hazards, including genes for known human mycotoxins.

## Results

### Genome sequencing and general features

**Figure 2:**
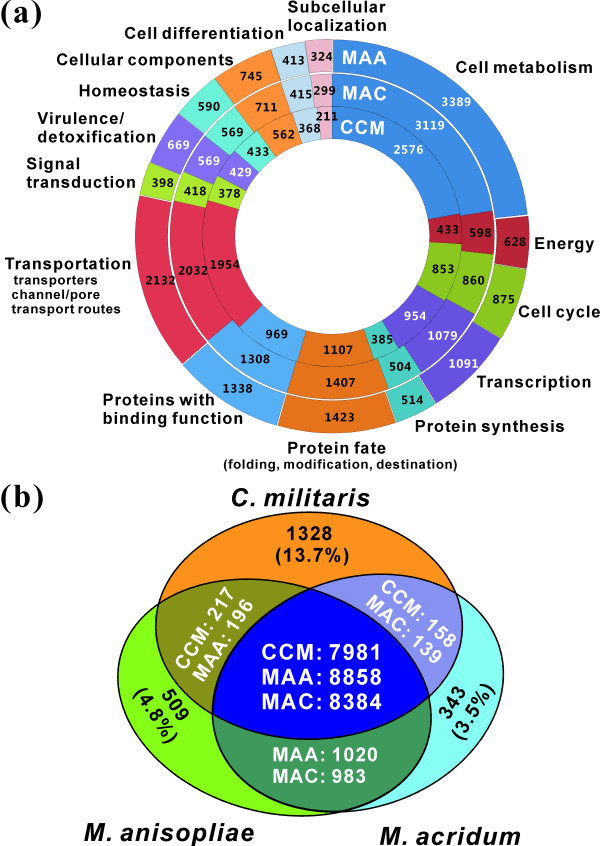
**Comparative genomics analysis of three insect pathogens**. **(a) **Functional classification and comparison of *C. militaris *(CCM), *M. anisopliae *(MAA) and *M. acridum *(MAC) proteins, showing that *C. militaris *has fewer genes in each category. Each circle represents the relative fraction of genes represented in each of the categories for each genome. **(b) **Reciprocal blast analysis of the predicted proteins among three insect pathogens. The cut-off E value is at ≤ 1e-5.

The *C. militaris *genome was shotgun sequenced to 147 × coverage and assembled into 33 scaffolds with an N50 of 4.6 Mb and a total genome size of 32.2 Mb. The genome is smaller than either the broad host range *Metarhizium anisopliae *(MAA) or the locust-specific pathogen *Metarhizium acridum *(MAC) that we sequenced previously (Table 1). The characteristic telomeric repeats (TTAGGG/CCCTAA)_n _were found at either 5' or 3' terminal of 13 scaffolds, including the terminal anchoring of two scaffolds, that is, the complete chromosomes. From mapping > 5,000 expressed sequence tags [12], the *C. militaris *genome was estimated to be > 99% complete. The genome was predicted to encode 9,684 protein genes, which is slightly fewer than *M. anisopliae *and *M. acridum *(Table 1). Consequently, many protein functional categories are smaller in *Cordyceps *than in *Metarhizium *spp. (Figure 2a). However, like *M. anisopliae *(17.6%) and *M. acridum *(15.1%), *C. militaris *has a higher proportion of its genes encoding putatively secreted proteins (15.9%) than other sequenced ascomycetes (5 to 10%) [10,13,14].

**Table 1:** Comparison of genome features among three insect pathogens

Features	*C. militaris*	*M. anisopliae*	*M. acridum*
Size (Mb)	32.2	39.0	38.1
Coverage (fold)	147 ×	100 ×	107 ×
Percentage G+C content	51.4	51.5	50.0
Percentage repeat rate	3.04	0.98	1.52
Protein-coding genes	9,684	10,582	9,849
Gene density (genes per Mb)	257	271	259
Exons per gene	3.0	2.8	2.7
Percentage secreted proteins	16.2	17.6	15.1
tRNA	136	141	122
Pseudogenes	102	363	440
NCBI accession	AEVU00000000	ADNJ00000000	ADNI00000000

Mb, mega base pairs.

An InterproScan analysis identified 2,736 conserved protein families in *C. militaris *(containing 6,725 proteins), fewer than those in *M. anisopliae *(7,556 proteins in 2,796 families) or *M. acridum *(6,948 proteins in 2,746 families) [11]. In particular, the number of transposases is much fewer in *C. militaris *(4) than in *Metarhizium *spp. (148 in *M. anisopliae *and 20 in *M. acridum*) or other sequenced ascomycetes (15 to 426) (Table S1 in Additional file 1). The *C. militaris *genome lacks retrotransposase (Table S2 in Additional file 1), and has more than three-fold fewer pseudogenes than *Metarhizium *spp. (Table S3 in Additional file 1). About 16% of the predicted *C. militaris *genes (1,547) are putatively involved in pathogen-host interactions; this proportion is slightly lower than for *Metarhizium *spp. (17.3% in MAA and 16.5% in MAC) but is higher than four plant pathogens (10.8 to 15.5%; *P *= 0.0476; false discovery rate (FDR) = 0.0152) (Table S4 in Additional file 1).

**Figure 3:**
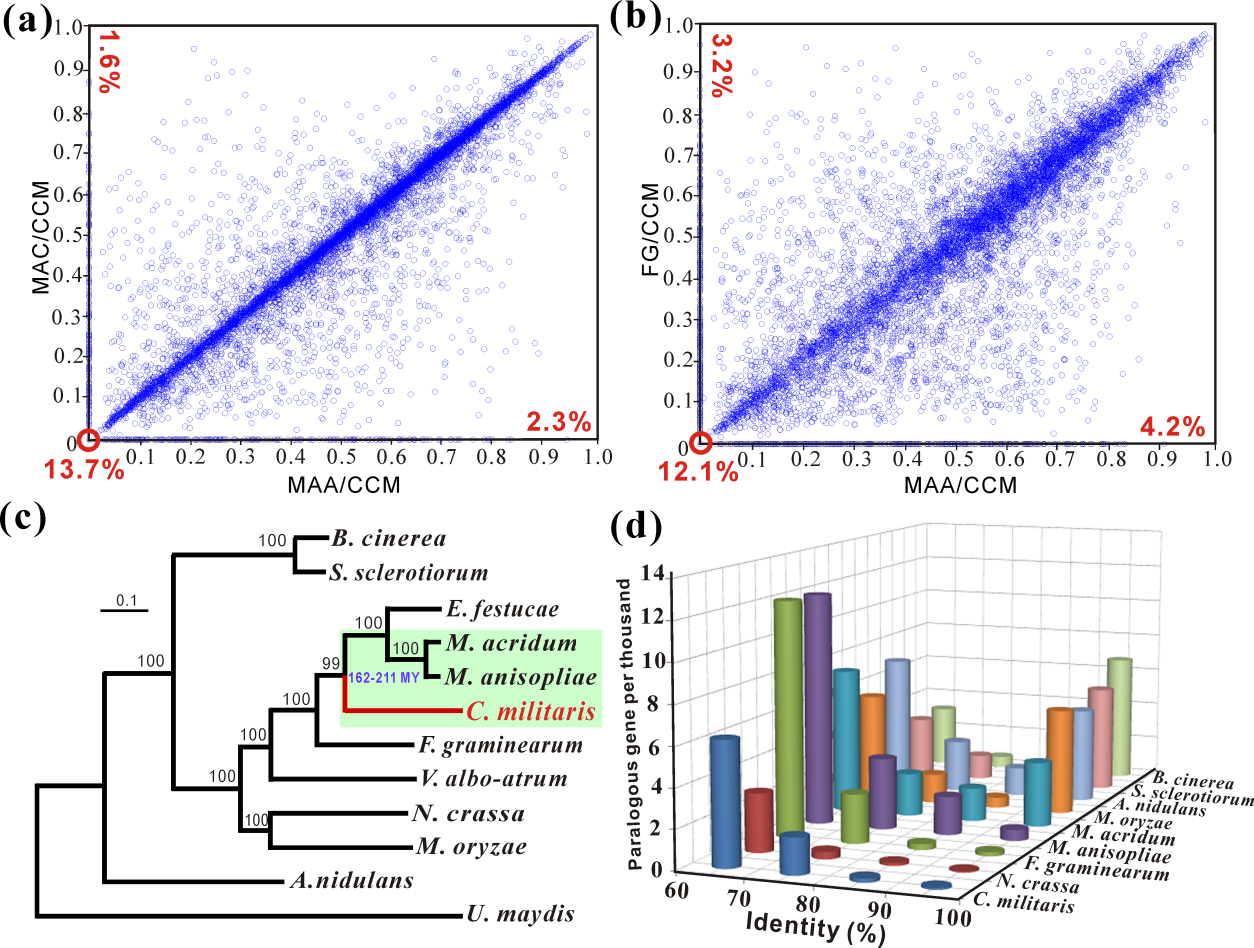
**Comparative genomics and evolutionary analysis of *C. militaris***. Scatter plots of Blast score ratio (BSR) analysis of (**a**) *C. militaris *(CCM), *M. anisopliae *(MAA) and *M. acridium *(MAC) genomes, and (**b**) CCM, MAA and *F. graminearum *(FG) genomes. The numbers in red at the lower left corners are the percentages of *C. militaris *species-specific sequences and the numbers at the upper left or lower right are the percentages of lineage-specific genes between pairs of genomes. (**c**) A maximum likelihood phylogenomic tree constructed using the Dayhoff amino acid substitution model showing the evolutionary relationship of *C. militaris *with different fungal species. Three insect pathogens are highlighted by the green shading. (**d**) Distribution of paralogous gene numbers with different levels of nucleotide similarity in *C. militaris *and other fungi. MY, million years.

More than 50% of *M. anisopliae *and *M. acridum *proteins have > 90% identity [11]. Although the rarely observed sexual stages of *Metarhizium *spp. have been identified as a *Cordyceps *species [1], the analysis revealed that < 2% of *C. militaris *genes were highly conserved in comparison with those from *Metarhizium *spp., that is, had Blast score ratio (BSR) values close to 1 (Figure 3a). A similar pattern was observed when comparing *C. militaris*, *M. anisopliae *and the plant pathogen *Fusarium graminearum *(Figure 3b). Comparative genomic analysis of the three insect pathogens found that the percentage of species-specific genes is much higher in *C. militaris *(13.7%) compared to *M. anisopliae *(4.8%) and *M. acridum *(3.5%) (Figure 2b). Based on the identities between orthologous proteins, *C. militaris *displays an average of approximately 63% amino acid identity with either *M. anisopliae *or *M. acridum*, slightly higher than with the plant pathogens *F. graminearum *(61.6%) and *Magnaporthe oryzae *(56.0%) (Table 2). Thus, the three insect pathogenic fungi are more highly diverged than *F. graminearum*, *Fusarium oxysporum *and *Fusarium verticillioides*, which share an average of 85% nucleotide sequence identity [14], and *Aspergillus nidulans*, *Aspergillus fumigatus *and *Aspergillus oryzae*, which share an average of 68% amino acid sequence identity [13], and *Trichoderma reesei*, *Trichoderma virens *and *Trichoderma atroviride*, which share an average of > 70% amino acid sequence identity [15].

**Table 2:** Genome-wide analysis of *C. militaris *gene sets.

Characteristics	*C. militaris*	**CMM core**^**a**^	**CMA restricted**^**b**^	**CMC restricted**^**c**^	CCM specific
Number of genes	9,684	7,981	217	158	1,328
Mean gene length (bp)	1,742	1,885	1,445	1,440	967
Mean number of introns per gene	1.99	2.05	1.77	1.69	1.71
Percentage genes without introns	21.3	20.1	22.6	28.5	27.5
Percentage GC content (excluding introns)	58.6	58.6	70.7	58.7	58.3
Number of InterproScan protein families	2,644	2,552	69	52	112
Number of secreted proteins	1,572	1,250	45	13	264
Number of PHI genes^d^	1,547	1,539	4	2	2
Number of TSA proteases	68	65	3	0	0
Number of MFS genes	245	242	0	2	1
Number of cytochrome P450s	57	56	0	1	0
Number of Pth11-like GPCRs	18	18	0	0	0
Number of protein kinases	167	167	0	0	0
Number of transcription factors	123	120	2	1	0
Number of glycoside hydrolases	105	103	2	0	0
Number of SM backbone genes	28	28	0	0	0
Number of horizontally transferred genes	49	30	5	1	12
Number of orthologs in *M. anisopliae*	6,863	6,705	158	NA	NA
Number of orthologs in *M. acridum*	6,762	6,644	NA	118	NA
Number of orthologs in *F. graminearum*	6,740	6,376	106	89	169
Number of orthologs in *M. oryzae*	6,219	5,937	90	80	112
Percentage identity to *M. anisopliae *orthologs	63.4	63.7	51.3	NA	NA
Percentage identity to *M. acridum *orthologs	63.4	63.6	NA	51.2	NA
Percentage identity to *F. graminearum *orthologs	61.6	62.3	51.9	52.8	46.7
Percentage identity to *M. oryzae *orthologs	56.0	56.4	48.3	48.0	45.3

^a^CMM core: *C. militaris *(CCM), *M. anisopliae *and *M. acridum *genes grouped with a cutoff E value of 1e-5 during reciprocal Blast analysis. ^b^CMA restricted: *C. militias *and *M. anisopliae *restricted genes grouped with a cutoff E value of 1e-5. ^c^CMC restricted: *C. militaris *and *M. acridum *restricted genes grouped at a cutoff E value of 1e-5. ^d^PHI genes, pathogen-host interaction genes predicted by blast analysis against the PHI database [72]. Identity was estimated at the amino acid level. GPCR, G-protein coupled receptor; MFS, major facilitator superfamily; NA, not available; SM, secondary metabolite; TSA, trypsin, subtilisin and aspartyl protease.

The regions containing at least three contiguous open reading frames that are not present in the reference genome are designated as genomic islands (GIs) [16]. Whole genome reciprocal analysis of three insect pathogens demonstrated that, in comparison to *Metarhizium *spp., *C. militaris *has 52 GIs (2% coverage of its genome, harboring 21% of its species-specific genes), which is many more than *M. anisopliae *(8 GIs, 0.3%) or *M. acridum *(5 GIs, 0.2%) when referenced to *C. militaris*. As in aspergilli [17], many *C. militaris *species-specific gene-encoding proteins do not have conserved domains and the genes are clustered together to form GIs (Table 2). A phylogenomic analysis established that the *Cordyceps *lineage is more closely related to the wheat pathogen *F. graminearum *(divergence time of 200 to 260 million years ago (MYA)) than it is to *Metarhizium *spp. (26 to 34 MYA) (Figure 3c). Thus, the lineage leading to *C. militaris *appears to have diverged from plant pathogens around the Triassic-Jurassic boundary (200 MYA), while *M. anisopliae *and *M. acridum *diverged after the Cretaceous Extinction Event (65 MYA) [18]. Analysis of paralogous genes found only one pair of *C. militaris *genes with > 90% nucleotide sequence similarities (Figure 3d), which is similar to *Neurospora crassa *(one pair) [13] and *F. graminearum *(two pairs) [19]. Analysis of 24 paired *C. militaris *genes showing > 70% nucleotide identities found a strong overall C:G to T:A mutation bias (Figure S1 in Additional file 2), consistent with repeat-induced point mutations, the DNA methylation-linked processes that cause mutations of repeated fungal sequences [15,20].

### Protein family analysis

We identified gene family expansions for proteases, chitinases, lipases and protein kinases in *C. militaris *when compared with phytopathogenic fungi, whereas gene family contractions occurred for glycoside hydrolases (GHs; *P *= 0.0144; FDR = 0.02), cutinases (*P *= 0.0065; FDR = 0.0226) and pectin lyases (*P *= 0.0245; FDR = 0.0284) (Table S1 in Additional file 1). The largest family expansions were for proteases. The *C. militaris *genome contains 61 families of proteases but most of them were included in families of serine proteases (180/381) and metallopeptidases (108/381) (Table S5 in Additional file 1). Gene expansions within the subtilisin (*P *= 0.0109; FDR = 0.0189) and trypsin (*P *= 0.0077; FDR = 0.0178) families are consistent with their being virulence factors in insect pathogens [11]. However, different families of proteases are expanded in *Metarhizium *spp. and *C. militaris*, consistent with each lineage 'reinventing the wheel' during the evolution of entomopathogenicity. Thus, relative to *Metarhizium *spp., the S01 trypsin and S08 subtilisin subfamilies are smaller and the S53 subfamily is larger (Table S6 in Additional file 1). The *C. militaris *genome has 12 trypsin genes compared to 4 or less in plant pathogens. It lacks four subfamilies of trypsins present in *M. anisopliae*. Interestingly, the bacterial-like chymotrypsin identified in *M. anisopliae *[21] is absent in *M. acridum *[11] but present as two copies in *C. militaris*. The A01 aspartyl proteases are virulence factors of both mammalian and plant pathogens because of their ability to cleave an array of host proteins [22]. Compared to phytopathogenic fungi (average 17), their number is significantly (*P *= 0.0059; FDR = 0.0057) expanded in the three insect pathogens (average 24) (Table S4 in Additional file 1).

Compared to many plant pathogens, *Metarhizium *spp. and *C. militaris *have fewer cutinases for degrading plant cell walls (Table S1 in Additional file 1). They also have fewer (average 137, *P *< 0.05) GHs than plant pathogens (average 199), including the lack of 20 GH families used by most plant pathogens and saprobes to target plant cell walls - for example, GH6, GH7 and GH61 cellulases, GH10 and GH11 xylanases, GH28 pectinases and GH78 rhamnosidases (Table S7 in Additional file 1). There are also significant differences in the spectrum of enzymes produced by the entomopathogens. For example, compared to *M. anisopliae*, *C. militaris *has few xyloglucosyl transferases (GH16) for xyloglucan catabolism and lacks α-glucuronidases (GH115) active on xylan oligomers or polymeric xylan [23]. Consistent with this, *C. militaris *grows very poorly on xylose when compared with *M. anisopliae *(Figure S2 in Additional file 2). A phosphoketolase MPK1 involved in pentose metabolism is required for full virulence of *M. anisopliae *[24], but the homolog is absent in *C. militaris*. However, GH18 chitinases similar to those used by *Metarhizium *to degrade insect cuticles [11] are well represented in the *C*. *militaris *genome (20 in CCM versus 30 in MAA and 19 in MAC) relative to plant pathogens (average 11) (Table S7 in Additional file 1).

Cytochrome P450s (CYPs) play essential roles in fungal physiologies, including detoxification, degradation of xenobiotics and the biosynthesis of secondary metabolites [25]. *C. militaris *has only about half as many CYPs as *Metarhizium *spp., and most other fungi (Table S8 in Additional file 1). Seventy CYP subfamilies present in *M. anisopliae *and/or *M. acridum *are absent in *C. militaris*. Of particular interest, *C. militaris *lacks CYP55, CYP58 and CYP65. CYP55 is a nitric oxide reductase required for denitrification [25]. Thus, unlike most filamentous fungi, *C. militaris *may not respond to hypoxia through the bacterial ammonia fermentation mechanism. The absence of CYP58 (trichodiene oxygenase) and CYP65 (trichothecene C-15 hydroxylase) suggests that *C. militaris *will not produce the mycotoxin trichothecene [26]. *M. anisopliae *can efficiently metabolize insect epicuticle alkanes [27]. The CYP52 subfamily for alkane hydroxylation [25] is well represented in *Cordyceps*.

The major facilitator superfamily (MFS) and ATP-binding cassette (ABC) transporters are the two biggest families of fungal transporters. Members of the former typically function as nutrient symporters and drug antiporters, whereas the latter are more often implicated in defense against toxic metabolites [28]. *C. militaris *has approximately half (123) the number of these transporters as *Metarhizium *(269 in MAA and 236 in MAC) (Table S9 in Additional file 1). The MFS transporters that are underrepresented in *Cordyceps *include the carbohydrate symporters (37 in CCM versus 48 in MAA, 51 in MAC and an average of 58 in plant pathogens), vitamin B2 (riboflavin) transporters (2 in CCM versus 17 each in *Metarhizium *species and an average of 4 in plant pathogens) and multidrug antiporters (23 in CCM versus 110 in MAA, 77 in MAC and an average of 10 in plant pathogens). Consistent with their having many multidrug transporters, *Metarhizium *spp. are resistant to diverse antibiotics and fungicides [29]. *Cordyceps *has more ABC-type drug and metal resistant proteins than *Metarhizium *and plant pathogens (63 in CCM, 56 in MAA, 51 in MAC and an average of 54 in plant pathogens). The amino acid and dipeptide transporters are similarly represented in the three insect pathogens and other fungi (46 in CCM versus 53 in MAA, 49 in MAC and an average of 45 in plant pathogens).

Fungal G-protein coupled receptors (GPCRs) are required for pheromone/nutrient sensing and host recognition [11]. Thus, the Pth11-like GPCR of *Magnaporthe *mediates cell differentiation in responses to plant inductive cues [30]. *C. militaris *has fewer GPCRs than *Metarhizium *spp. and is particularly impoverished in Pth11-like GPCRs (Table S10 in Additional file 1). *C. militaris *has a similar number (167) of protein kinases as *M. anisopliae *(161) but less than *M. acridum *(192) (Table S11 in Additional file 1). Like other fungi, fungal specific transcription factors (TFs) and zinc finger TFs represent the two largest classes of TFs in *C. militaris *and their numbers are similar to those of other fungi (Table S1 in Additional file 1).

### Mating-type and sexuality analysis

**Figure 4:**
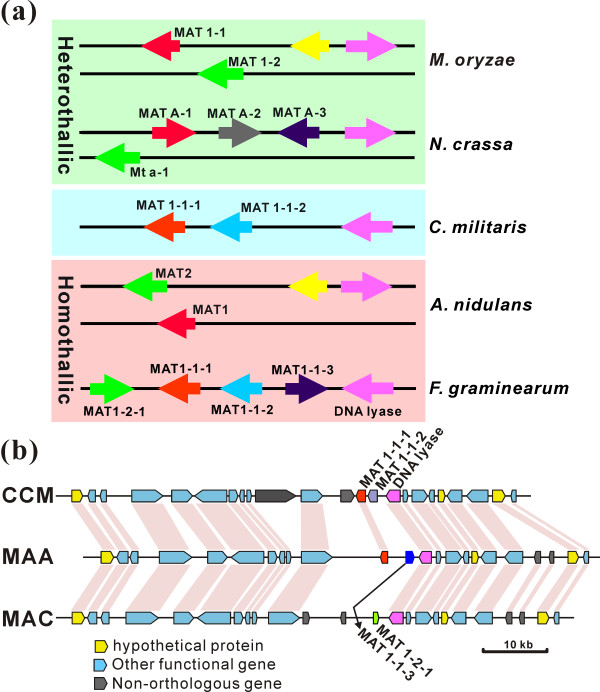
**Comparative analysis of the *C. militaris *mating-type (MAT) locus**. (**a**) Comparative analysis of the *C. militaris *MAT locus with those of sexually heterothallic and homothallic fungal species. Genes labeled in the same color have orthologous relationships. (**b**) Syntenic relationship of the MAT loci and their flanking regions between the three insect pathogens *C. militaris *(CCM), *M. anisopliae *(MAA) and *M. acridum *(MAC).

The fruiting bodies of *Cordyceps *spp. are the most commonly sold traditional Chinese medicine products [5]. However, the sexual cycle and fruiting of *C. militaris *is poorly understood. We only identified a MAT1-1 mating-type locus, including MAT1-1-1 and MAT1-1-2 genes, in the sequenced Cm01 strain, suggesting that *C. militaris *is heterothallic (Figure 4a). A single mating-type locus was also found in *M. anisopliae *(MAT1-1) and *M. acridum *(MAT1-2). Like aspergilli [10], the idiomorphic regions of the three insect pathogens are highly divergent (Figure 4a). The MAT1-1 locus of *M. anisopliae *contains a MAT1-1-3 gene but lacks the MAT1-1-2 gene present in *C. militaris*. Except for the mating-type locus region, most *A. nidulans *and *N. crassa *genes involved in mating, fruiting, karyogamy and meiosis are also present in insect pathogens (Table S12 in Additional file 1).

**Figure 5:**
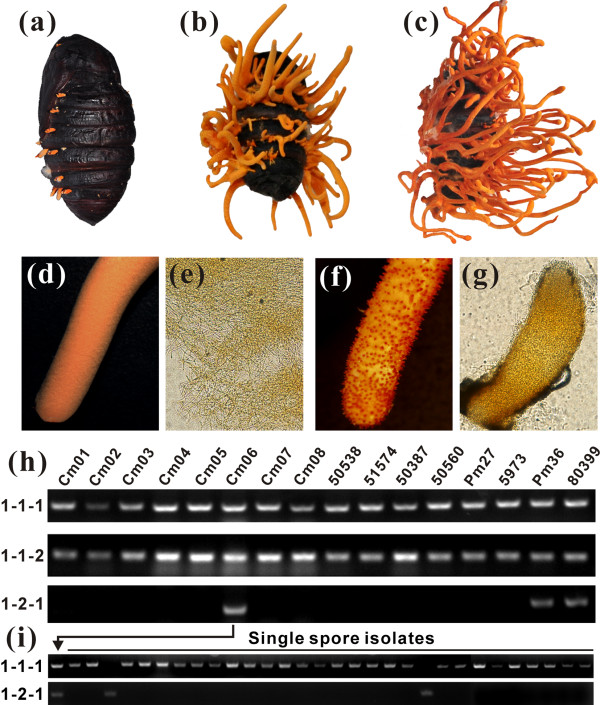
**Fruiting body development, sexuality and mating-type analysis**. **(a-c) **Chinese Tussah silkmoth pupae were inoculated with conidia from the *C. militaris *Cm01 strain and incubated for 14 days (a), 29 days (b) and 59 days (c) to produce nascent, mid-term and developmentally mature fruiting bodies. **(d-g) **The mature fruiting bodies of the Cm01 strain do not produce perithecia (d, e) but those of strain Cm06 are completely covered with protruded perithecia (f, g). **(h) **PCR examination of different strains (numbers labeled on the top) showed that strains Cm06, Pm36 and 80399 contain the MAT1-1-1, MAT1-1-2 and MAT1-2-1 genes while Cm01 and other strains lack the MAT1-2-1 gene. **(i) **PCR examination of 30 randomly selected single spore isolates from the hybrid strain Cm06 showed that only 2 out of 30 isolates contain the MAT1-2-1 gene.

**Figure 6:**
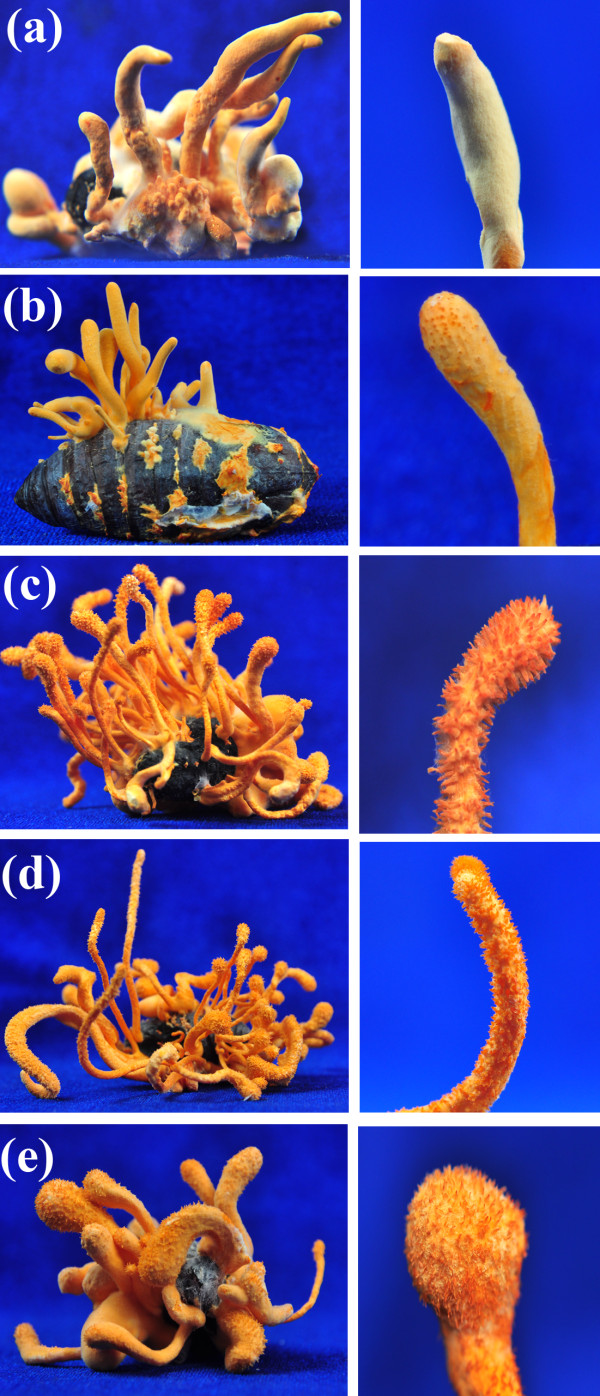
**Fruiting structures of different mating-type isolates**. **(a, b) **Sterile fruiting bodies formed on caterpillar pupae after inoculation of MAT1-1 (a) and MAT1-2 (b) isolates acquired by single conidial spore isolation from a MAT1-1/MAT1-2 hybrid strain, Cm06. (**c-e**) Fertile fruiting structures formed on caterpillar pupae after inoculation of the mixed conidia of MAT1-1 (Cm01) and MAT1-2 (Cm06) at ratios of 1:9 (c), 1:1 (d) and 9:1 (e), respectively. The right panels represent close-up views of corresponding sterile (without protruded perithecia) or fertile (with protruded perithecia) fruiting bodies. After inoculation, the pupae were incubated at 22°C with a 12:12 hour light:dark cycle for 60 days.

Strain Cm01 forms fruiting bodies on caterpillar pupae that lack perithecia and ascospores (Figure 5a-e). Thus, it is the first ascomycete species reported to fruit without an opposite mating-type partner. Other *C. militaris *isolates could also fruit sterilely with a single mating-type locus (Figure 6a, b). However, a hybrid strain, Cm06, with both MAT1-1 and MAT1-2 loci produced sexual perithecia and ascospores (Figure 5f, g). In addition, the sexual structures could be similarly re-formed after inoculation of the caterpillar pupae with different ratios of MAT1-1 and MAT1-2 isolate conidia (Figure 6c-e), confirming that *C. militaris *is heterothallic. PCR examination of 18 field-collected strains identified three containing both MAT1-1 and MAT1-2 loci (Figure 5h). However, 28 out of 30 single spore isolates of the Cm06 strain belonged to the MAT1-1 mating-type (Figure 5i). A similar unequal prevalence of mating types occurs in the dermatophyte fungus [31].

### Metabolism of medically active components and mycotoxins

**Figure 7:**
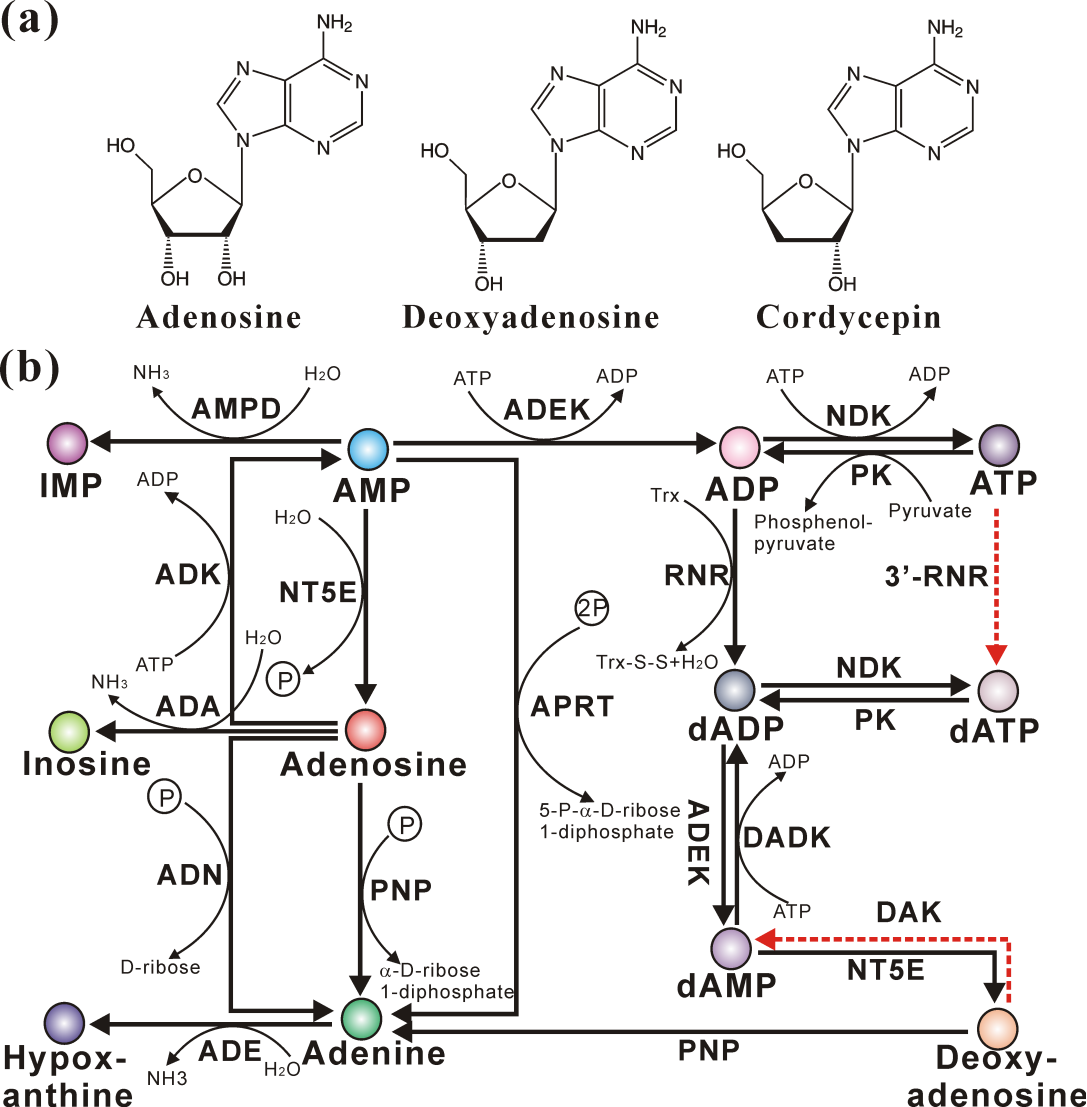
**Cordycepin analogues and the *C. militaris *adenine metabolic pathway**. **(a) **The structures of cordycepin analogues. **(b) **The *C. militaris *adenine metabolic pathway. Abbreviations for different enzymes: ADA, adenosine deaminase; ADE, adenine deaminase; ADEK, adenylate kinase; ADK, adenosine kinase; ADN, adenosine nucleosidase; AMPD, AMP deaminase; APRT, adenine phosphoribosytransferase; DADK, deoxyadenylate kinase; DAK, deoxyadenosine kinase; NDK, nucleoside-diphosphate kinase; NT5E, 5'-nucleotidase; PK, pyruvate kinase; PNP, purine nucleoside phosphorylase; 3'-RNR, ribonucleotide triphosphate reductase. The red dashed lines show metabolic pathways present in other organisms but absent in *C. militaris*.

One of the main pharmaceutically active components of *C. militaris *is cordycepin [5,6], which is structurally similar to 2'-deoxyadenosine (Figure 7a). *C. militaris *possesses most of the genes required for metabolism of adenine and adenosine except for lacking a ribonucleotide trisphosphate reductase (RNR; converts ATP to dATP) and a deoxyadenosine kinase (converts deoxyadenosine to dAMP) (Figure 7b; Table S13 in Additional file 1). It has been suggested that the biosynthesis of cordycepin proceeds through a reductive mechanism as described for the formation of 2'-deoxyadenosine [32]. However, *C. militaris *resembles *Metarhizium *and other cordycepin non-producing fungi in having only two highly conserved subunits of class I RNRs (Figure S3 in Additional file 2). The substrates for class I RNRs are ADP, GDP, CDP and UDP but not TDP or nucleosides, and as the reductive reaction proceeds via a free radical mechanism [33], *C. militaris *RNRs will not be involved in cordycepin production.

**Figure 8:**
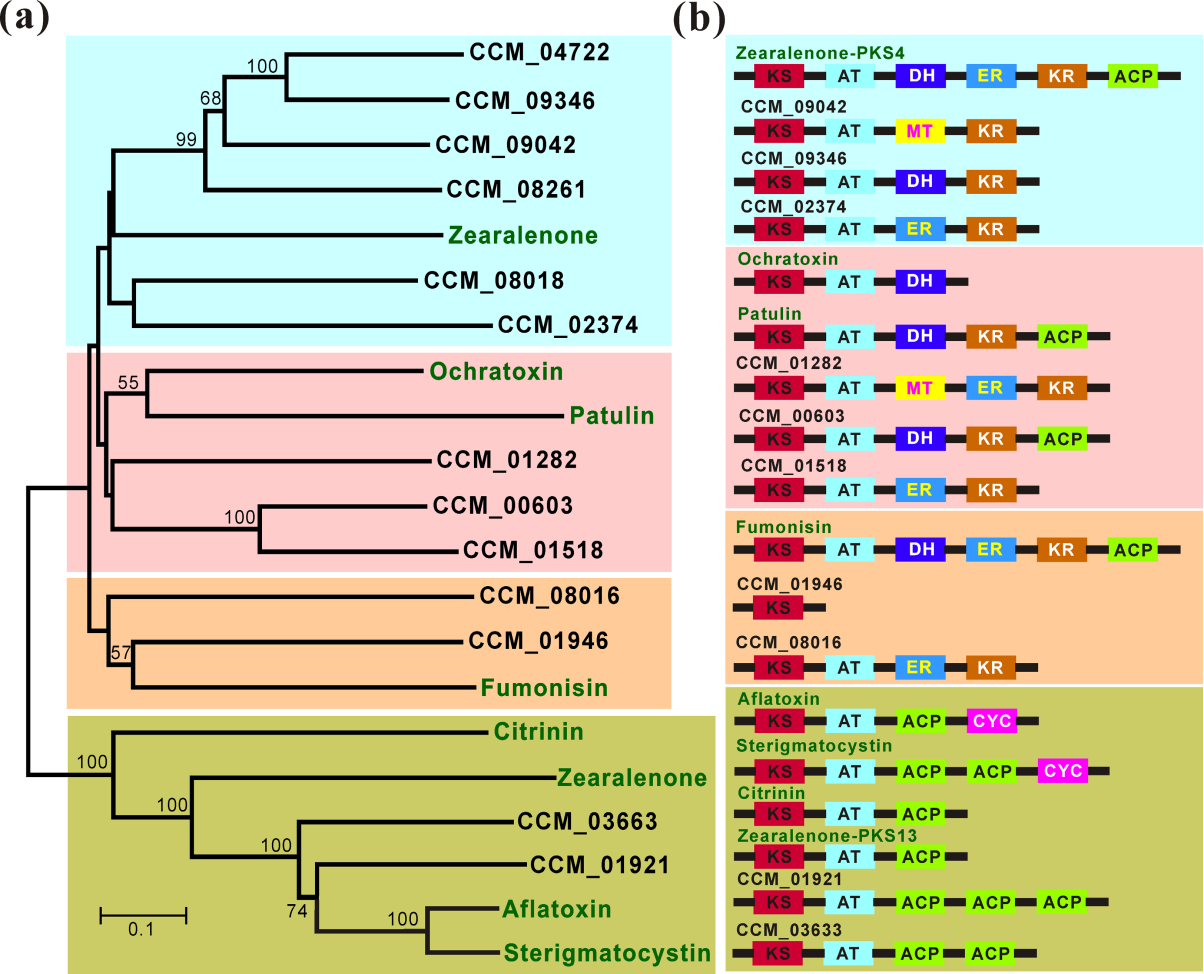
**Phylogenetic and modular analysis of *C. militaris *polyketide synthases compared with those involved in the production of human mycotoxins**. (**a**) A neighbor-joining tree showing the relationships of ketoacyl CoA synthase (KS) domain sequences. (**b**) Modulation and comparison of *C. militaris *PKSs with those involved in production of mycotoxins. The PKS-NRPS hybrid proteins CCM_04722, CCM_08261 and CCM_08018 are not included in the analysis. Domain definitions: ACP, acyl carrier protein domain; AT, acyltransferase domain; CYC, cyclase domain; DH, dehydratase domain; ER, enoyl reductase domain; KR, ketoreductase domain; MT, methyltransferase domain; TE, thioesterase domain. The accessions and references for different mycotoxins are provided in the Materials and methods.

Contamination of food and feed by mycotoxins is a longstanding threat to the health of humans and animals [26]. *C. militaris *has been consumed for hundreds of years, implying safety, but the genome data allowed us to make the first comprehensive inventory of *Cordyceps *genes involved in biosynthesis of secondary metabolites for comparison with known mycotoxins. There are fewer secondary metabolite core genes in *C. militaris *relative to *Metarhizium *spp. or plant pathogens (Table 3). In comparison to *Metarhizium *spp., *Cordyceps *has fewer terpenoid synthases, polyketide synthases (PKSs) and non-ribosomal peptide synthetases (NRPSs). Phylogenetic analysis of *Cordyceps *PKS and PKS-like genes using the ketoacyl CoA synthase (KS) domain sequences found that the *C. militaris *proteins grouped into different clusters compared to PKSs for known mycotoxins (Figure 8a). In addition, modular analysis indicated that, except for CCM_00603, which has a similar domain organization to the *Aspergillus clavatus PatK *gene for patulin biosynthesis, *C. militaris *PKSs are structurally different from mycotoxin PKSs (Figure 8b). The further survey showed that the CCM_00603 protein has only 27% identity with PatK and the gene cluster for patulin biosynthesis is absent in *C. militaris *(Table S14 in Additional file 1). This suggests that *C. militaris *PKSs do not produce patulin or other known human mycotoxins. Similarly, phylogenetic and modular analyses indicated that *Cordyceps *NRPSs had different protein structures than any NRPSs involved in production of known mycotoxins like enniatin, HC-toxin and gliotoxin (Figure S4 in Additional file 2).

**Table 3:** Numbers of core genes involved in the biosynthesis of secondary metabolites in different fungi

Core gene	CCM	MAA	MAC	FG	MO	BC	SS	NC	AN
DMAT	1	5	3	0	3	1	1	1	6
TC	3	3	3	3	3	3	3	3	5
TS	2	8	6	11	8	7	1	2	5
FAS	1	2	2	1	1	1	1	1	1
GGPS	3	4	4	3	3	0	0	1	0
NRPS	5	14	13	10	5	6	5	3	11
NRPS-like	8	9	8	11	6	8	5	3	12
PKS	9	24	13	14	12	16	16	6	24
PKS-like	2	3	4	1	3	6	2	2	4
HYBRID	3	5	1	1	3	0	0	0	1
Total	37	77	57	55	47	48	34	22	69

Core genes encoding: DMAT, dimethylallyl tryptophan synthase; TC, terpenoid cyclase; TS, terpenoid synthase; FAS, fatty-acid synthase; GGPS, geranylgeranyl diphosphate synthase; NRPS, non-ribosomal peptide synthetase; PKS, polyketide synthetase; HYBRID, hybrid PKS-NRPS enzyme. Fungal species: CCM, *C. militaris*; MAA, *M. anisopliae*; MAC, *M. acridum*; FG, *F. graminearum*; MO, *M. oryzae*; BC, *Botrytis cinerea*; SS, *Sclerotinia sclerotiorum*; NC, *N. crassa*; AN, *A. nidulans*.

The mycotoxin ergot alkaloids have a wide range of biological activities and are important in pharmaceuticals and agriculture [26]. Dimethylallyl tryptophan synthase (DMAT) catalyzes the alkylation of L-tryptophan, the first committed step in the ergot alkaloid biosynthetic pathway [34]. *C. militaris *has one putative DMAT gene (CCM_04410), in contrast to five in *M. anisopliae *and three in *M. acridum *(Table 3). A phylogenetic analysis showed that CCM_04410 is not clustered with the *Claviceps *DMAT clade involved in ergot alkaloid production (Figure S5 in Additional file 2). The trichothecenes T-2 toxin and deoxynivalenol (type B trichothecene) are natural fungal products that are toxic to both animals and plants [35]. Consistent with lacking CYP58 and CYP65, the *C. militaris *genome also lacks trichodiene synthase (Table S15 in Additional file 1). Thus, unlike *Fusarium *[26], *C. militaris *is not predicted to produce trichothecene mycotoxins. The presence of terpenoid cyclase, terpenoid synthase, fatty-acid synthase and geranylgeranyl diphosphate synthase genes in the *C. militaris *genome suggests that the fungus is capable of producing an array of metabolites, but the identity of these and their biological activities remain to be determined.

### Transcriptional regulation of fruiting body development

**Figure 9:**
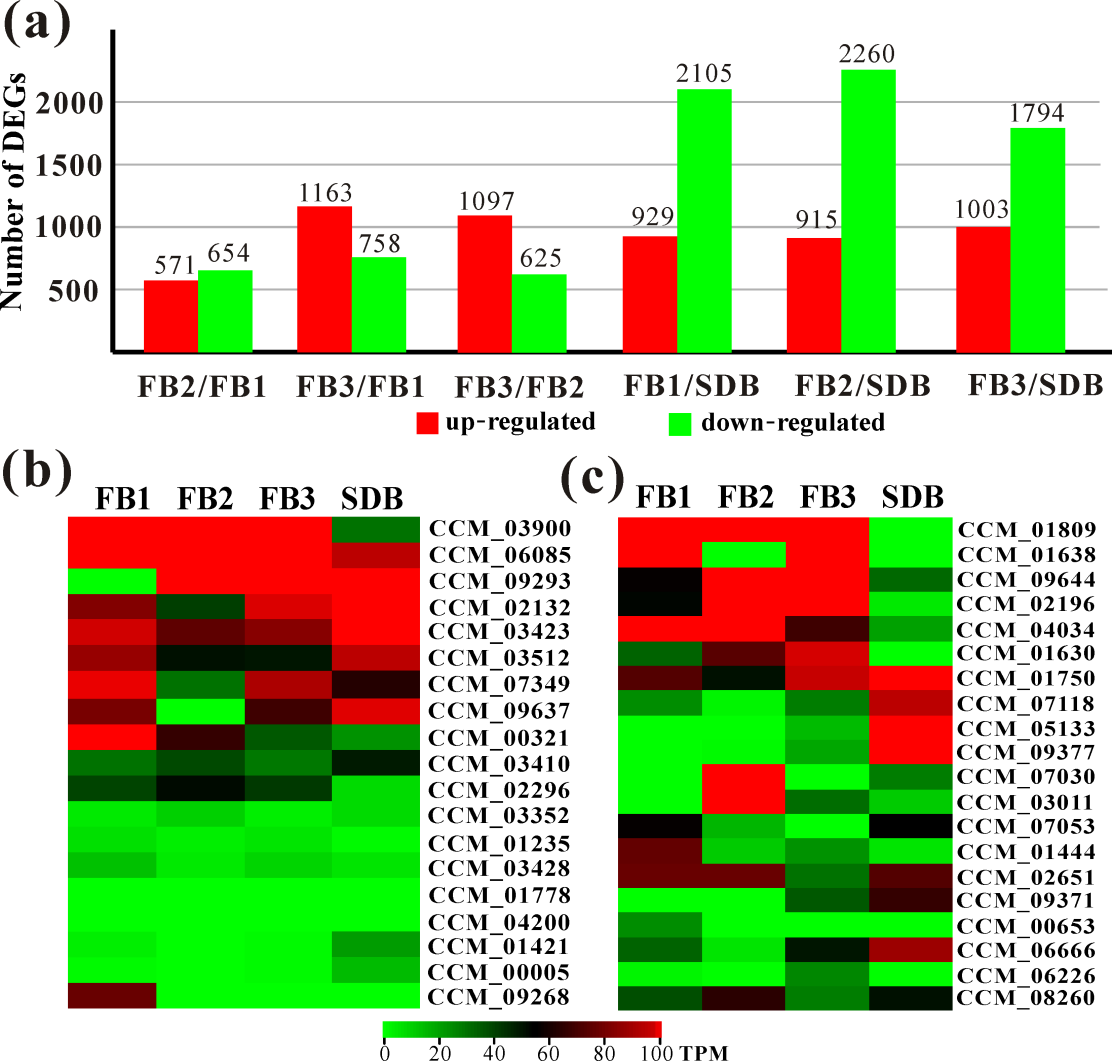
**Differential gene expression by *C. militaris *in association with fruiting structure formation or growth in a liquid medium**. (**a**) Estimation of significantly up- and down-regulated genes between different samples. (**b) **Heat map of protein kinases associated with the mitogen-activated and cAMP-dependent protein kinase pathways at different developmental stages. (**c**) Heat map of the highly expressed transcription factors at different developmental stages. Genes with expression values > 100 transcripts per million tags (TPM) are also indicated in red. Annotation information for the genes is provided in Table S19 in Additional file 1. DEG, differentially expressed gene. FB1, FB2 and FB3 are associated with nascent, stalk formation and mature developmental stages shown in Figure 5a-c, respectively. The transcriptome of undifferentiated mycelia harvested from SDB was included as a reference for gene expression analysis.

To identify genes associated with *C. militaris *fruiting body development, we compared the expression profiles of undifferentiated mycelia from Sabouraud dextrose broth (SDB) culture with developmental stages on caterpillar pupae defined as nascent (14 days, termed as sample FB1), stalk formation (29 days, FB2) and mature fruiting bodies (59 days, FB3) (Figure 5a-c). Of the 9,684 genes, more than 63% were expressed during both undifferentiated hyphal growth and formation of fruiting bodies (Table S16 in Additional file 1). Relative to the growth in SDB, more than 900 genes were significantly (*P *< 0.05; FDR < 0.001) up-regulated while around 2,000 genes were down-regulated during fungal fruiting (Figure 9a). A Pearson correlation analysis indicated that transcriptional profiles at the different stages of fruiting body formation more closely resembled each other than they resembled the transcriptomes of undifferentiated mycelia (Figure S6a in Additional file 2). This is consistent with a Venn diagram analysis of the commonest co-expressed genes between different samples (Figure S6b in Additional file 2). Of the 100 most highly expressed genes in developing *C. militaris *fruiting bodies, 26 (FB1), 31 (FB2) and 37 (FB3) are functionally uncharacterized (Table S17 in Additional file 1). This suggests that the genes with unknown function are more likely to be stringently regulated and involved in developmental processes than orthologs of genes with known function. These genes are thereby the targets for future functional studies. In general, the genes involved in cell wall structure and biogenesis, detoxification, protein degradation and amino acid transportation were significantly up-regulated during formation of fruiting structures. In contrast, most of the genes specifically up-regulated by undifferentiated SDB cultures were involved in rapid growth and carbohydrate metabolism. Concomitant with fruiting structure maturation, the genes for cytoskeletal organization, cell cycle and secondary metabolism were up-regulated.

Unlike other fungi, *C. militaris *can fruit sterilely in the absence of a sexual partner (Figure 5a-c). Perhaps because of this, 31 of the 42 *C. militaris *orthologs of sex-related genes identified in other ascomycetes were not expressed or transcribed at low levels (< 10 transcripts per million tags (TPM)) in sterile fruiting bodies (Table S18 in Additional file 1). However, in some cases, *C. militaris *expresses paralogous genes to those employed by other fungi, suggesting they have co-opted different components of the same signal transduction pathways to fulfill similar functions. For example, GATA-type TFs are important for fruiting in both *A. nidulans *and *N. crassa *[36], but *C. militaris *fruiting structures expressed orthologs of these genes at very low levels or not at all (Table S19 in Additional file 1). In contrast, the Zn2Cys6-type TFs were highly transcribed during fruiting but not in undifferentiated fungal mycelia - for example, CCM_01809 and CCM_09644 (Figure 9b) - indicating that Zn2Cys6 type TFs are predominately involved in the major developmental switch of production of fruiting structures.

**Figure 10:**
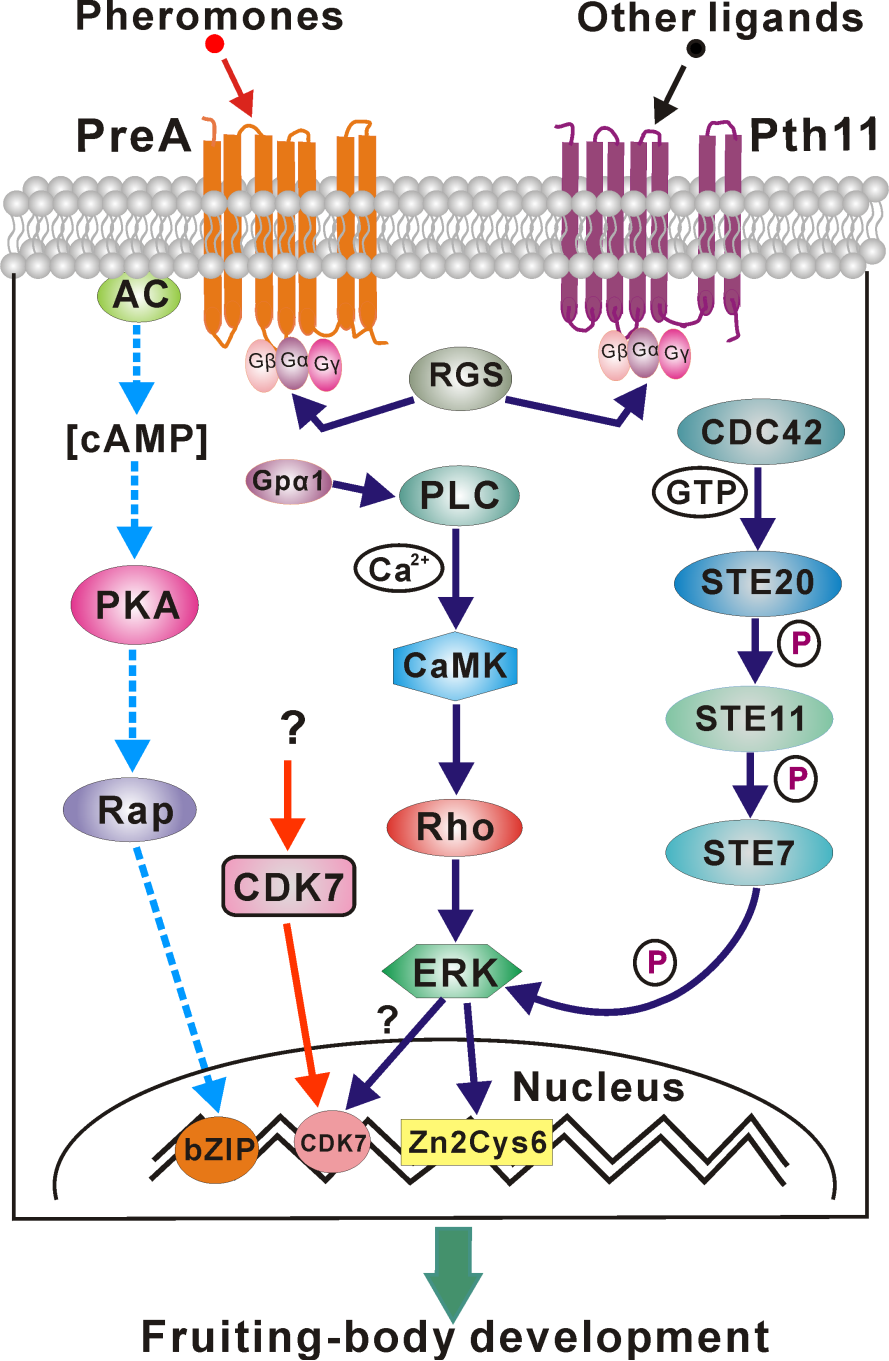
**Putative signal transduction pathways regulating fruiting body development in *C. militaris***. The dashed lines show the cAMP-dependent PKA pathway, which might not be involved in control of fruiting in *C. militaris*. The transcription data for different components are provided in Table S19 in Additional file 1. AC, adenylate cyclase; CaMK, calmodulin-dependent protein kinase; CDK, cyclin-dependent kinase; PLC, phospholiapse C; RGS, regulator of G protein signaling.

Pheromone receptors, that is, GPCRs, control fungal fruiting body formation and sexual cycle but not vegetative growth [36]. The pheromone receptor of *C. militaris *has not been identified. In comparison to undifferentiated mycelial growth, a putative pheromone receptor (CCM_01499) and a Pth11-like GPCR (CCM_03015) were significantly up-regulated (*P *< 0.05, FDR < 0.001), respectively, during initiation of fruiting body formation. Mitogen-activated protein kinase (MAPK) genes are required for fruiting in *Aspergillus *(AN1017) and *Neurospora *(NC02393) [36], but orthologous genes were not transcribed (CCM_04200 versus AN1017) or transcribed at low levels (CCM_01235 versus NCU02393) by *C. militaris *(Table S19 in Additional file 1). However, *Cordyceps *sharply up-regulated (*P *< 0.05, FDR < 0.001) a MAPK paralog (CCM_09637) as well as a calcium regulated kinase (CaMK, CCM_06085) (Figure 9c). These data, taken in conjunction with the single adenylate cyclase (CCM_06928) not being transcribed and the low level expression of both protein kinase A (PKA; CCM_03352) and Rap GTPase (CCM_01391), indicate that fruiting by *C. militaris *in the absence of a partner is more dependent on the MAPK pathway than the cAMP-dependent PKA pathway (Figure 10).

## Discussion

We report here the first genome analysis of a *Cordyceps *species, the medicinal lepidopteran pathogen *C. militaris*, and show that the fungus is capable of fruiting without an opposite mating-type partner. We also show that it lacks genes known to be involved in production of human mycotoxins. Being an insect pathogen, the *C. militaris *genome contains thousands of genes putatively involved in interactions with insect hosts. *Cordyceps *resembles *Metarhizium *spp. in having a very high percentage of secreted proteins relative to plant pathogens and saprophytes and expanded families of proteases and chitinases with targets in insect hosts. However, insect-killing strategies may differ between *Cordyceps *and *Metarhizium *due to differences in gene content. Mating-type analysis indicated that sexual reproduction in *C. militaris *is heterothallic. Transcriptional profiling indicated that fruiting of the MAT1-1 *C. militaris *strain involves induction of the MAPK pathway, but unlike other homothallic or heterothallic fungi, the PKA pathway was not up-regulated. It remains to be determined whether this reflects the very unusual ability of *C. militaris *to produce fruiting bodies without an opposite mating-type partner.

Aside from knowing that *C. militaris *infects lepidopteran pupae [37], the life cycle of *C. militaris *in nature is poorly understood [8]. Following disease, survival in soil may depend on the sexual stage of *Cordyceps *providing resilient long-lived ascospores as described in other fungi [38]. Micro-cycle conidiation from germinated ascospores could adapt the fungus to nutrient poor niches (Figure 1). *Metarhizium *does not produce ascospores but flourishes in plant rhizospheres, which thus provide an alternative habitat in the absence of insect hosts. *C. militaris *can grow on germinated soybeans [39], suggesting a potential for an association with plants. However, relative to *Metarhizium *and most other ascomycetes, many protein families are smaller in the *C. militaris *genome, especially serine proteases, GHs, CYPs, MFS transporters and signal transduction factors. These families would be involved in scavenging for nutrients, avoidance of host defenses and toxins and other processes related to pathogenicity and a saprobic lifestyle. Around two-thirds of these protein families include pathogen-host interaction genes in plant-associated fungi (Table S4 in Additional file 1). Further studies on the ecology of *Cordyceps *spp. will shed more light on the relevance of the *C. militaris *genome to the evolution of gene families in relation to acquisition/loss of capability for dual plant/insect colonization and host range specialization.

The phylogenomic analysis demonstrated that the lineage leading to *Cordyceps *spp. diverged after most well known plant pathogens, including *F. graminearum*, but 130 MYA before *Metarhizium *diverged from the grass endophyte *Epichloë festucae*. The estimate of a Triassic-Jurassic boundary origin for the *Cordyceps *lineage and the post-Cretaceous origin of *Metarhizium *spp. is consistent with the hypocrealean fungi of Cordycipitaceae (includes *Cordyceps *spp.), Clavicipitaceae (includes *Metarhizium *spp.) and Ophiocordycipitaceae splitting about the same time as insects and angiosperms were diversifying [40]. Families of proteases and chitinases are not expanded or lost in the *E. festucae *genome as they are in *Cordyceps *and *Metarhizium*, exemplifying convergent evolution to insect pathogenicity. Besides proteases and chitinases, experimentally verified *Metarhizium *virulence-associated genes with homologs in the *C. militaris *genome include a perilipin-like protein (CCM_06103 versus MAA_08819) to control cellular lipid storage and appressorium penetration [41], and an osmosensor (CCM_04885 versus MAA_01551) to mediate adaptation to the insect hemocoel [42]. Homologs of these genes are broadly distributed among ascomycetes, indicative of an ancient origin. However, *Cordyceps *lacks other *Metarhizium *pathogenicity-related genes, including a collagen-like protein to evade the host immune system [43], a phosphoketolase for pentose metabolism [24] and the adhesins to mediate spore adhesions to insect and plant surfaces [44]. The absence of key components of the *Metarhizium *entomopathogenicity 'toolkit' from *C. militaris *indicates that it has evolved different determinants to mediate its interactions with insects.

Like *N. crassa *and *F. graminearum*, *C. miltaris *lacks highly similar paralogs, a hallmark of the repeat-induced point mutation (RIP) mechanism [20]. *C. militaris *has an ortholog (CCM_03609) of the *N. crassa *RIP defective gene (NCU02034), a cytosine methyltranserase essential for RIP [45]. The high C→T and G→A mutation bias in the *C. militaris *genome and the readiness of *C. miltaris *to undergo the sexual cycle suggests that RIP is commonplace in *C. militaris *like many ascomycetes [10,15,19]. Since RIP can function effectively against selfish DNAs [46], it likely contributes, at least in part, to *C. militaris *having few DNA type transposon encoded genes, that is, transposases [15].

There are many more orphan genes in *Cordyceps *than in *Metarhizium *spp., underscoring that much about the proteome of *Cordyceps *spp. remains unknown. It is speculated that orphan genes arise from gene duplication, shuffling of gene fragments, mobile element effects, mutation of existing sequences, horizontal gene transfer and *de novo *origination from non-coding DNAs [47]. *De novo *creation of new genes is probably rare [48]. A role for mobile element effects is also unlikely given how few putative transposase genes are present in the *C. militaris *genome. Putative horizontal gene transfer genes are even fewer in *Cordyceps *than in *Metarhizium*. Thus, the numerous orphans in the *C. militaris *genome most likely arose from frequent mutations caused by RIP in existing (duplicated) sequences. Just as the *Metarhizium*-specific collagen-like protein is essentially required to camouflage cells from host immune recognition [43]. The transcriptome data showed that 428 of the 1,329 orphan genes were transcribed during fruiting. Of the 100 most highly expressed genes in developing *C. militaris *fruiting bodies, about one-third are orphans (Table S17 in Additional file 1), underscoring the potential of orphans to have specific functions. Likewise, genes that are apparently unique to the mushroom *Schizophyllum commune *are more likely to be expressed during mushroom formation [49].

Concern has been raised about the possibility of harmful side effects of traditional Chinese medicines, including *Cordyceps *[50]. Consistent with genotoxicity and cytotoxicity assays that show *Cordyceps *products to be safe for consumption [51], there is no evidence in the *C. militaris *genome for genes involved in the production of known mycotoxins. However, safety could only be completely verified by meticulous profiling of the metabolites produced by the fungus under diverse growth conditions. The *C. militaris *genome data will facilitate these processes as well as help with elucidation of the biosynthetic pathways of different metabolites.

The analysis of *C. militaris *genome indicates that it is sexually heterothallic, but strikingly, both the MAT1-1 single mating-type and MAT1-1/MAT1-2 hybrid strains can form fruiting bodies, which means that *C. militaris *is capable of fruiting without a partner. Single mating-type (haploid) fruiting has also been observed in the human pathogens *Cryptococcus neoformans *and *Candida albicans *[52]. Given that perithecia and ascospores are not produced by MAT1-1 fruiting bodies, *C. militaris *haploid fruiting is different from the same-sex mating and fruiting of *C. neoformans*, in which diploidization and meiosis can occur. In budding yeast, meiotic recombination is initiated by the formation of double-strand breaks catalyzed by SPO11, a meiosis-specific endonuclease [53]. The meiosis-specific recombinase DMC1 and the DNA repair enzyme RAD51 then co-localize to double-strand breaks and function together for meiotic recombination [54]. The *C. militaris *homologue of yeast SPO11 (CCM_09527) was up-regulated more than five-fold during fruiting body maturation (the TPM ratio of FB2/FB1 = 8.1; FB3/FB2 = 5.7). Intriguingly, the *C. militaris *genome lacks a yeast RAD51 ortholog, but its DMC1 ortholog (CCM_06822) contains a RAD51 domain. CCM_06822 was not expressed by *C. militaris *during fruiting, which may explain why the *C. militaris *MAT1-1 strain forms fruiting bodies without meiosis. Consistent with this, a putative cyclin dependent kinase 7 (CDK7; CCM_03900) was up-regulated during fungal fruiting (Table S19 in Additional file 1). Orthologs of CDK7 initiate DNA synthesis and facilitate mitosis instead of meiosis [55].

## Conclusions

In conclusion, we report on the genome sequencing, comparative genome analysis and transcriptional regulation of fruiting body development in the medicinal fungus *C. militaris*. The sequence data should markedly enhance the pace of molecular research on *Cordyceps *biology, fungal sex and pathogenicity, and will have impacts on the commercial production of fruiting structures. The genomic sequence will also be an essential tool to unravel the mechanisms by which *C. militaris *produces medicinal compounds and so further their exploitation.

## Materials and methods

### Fungal strains

*C. militaris *strain Cm01 (CGMCC 3.14242) was selected for genome sequencing as it is culturally stable and commercialized in China. The culture was maintained either on artificial medium or silkworm pupae as previously described [12]. Several different *C. militaris *strains were included in this study for PCR genotyping of mating-type genes (Figure 5h).

### Genome sequencing and assembly

The genome of *C. militaris *strain Cm01 was shotgun sequenced using a Roche 454 GS FLX system for massively parallel pyrosequencing for 2.25 runs at the Chinese National Human Genome Center (Shanghai, China). This resulted in 951 Mb of sequence data (29.6 × coverage) with an average read length of 385 bp. Assembly was performed using the Newbler software (v2.3) within the Roche 454 suite package [56], which produced 597 contigs with a total size of 32.2 Mb. For sequence scaffolding, a DNA library of 2- to 5-kb inserts was generated and sequenced with an ABI SOLiD system (Carlsbad, California, USA). This resulted in 3.8 Gb of mate-pair reads (117.4 × coverage) to improve sequence quality and construct scaffolds. By mapping the reads to contigs, 578 contigs were assembled into 13 scaffolds and 19 contigs less than 2 kb left outside. The raw data of 454 and SOLiD reads have been deposited at NCBI's Sequence Read Archive under accession number SRA047932 and the whole project has been deposited at DDBJ/EMBL/GenBank under accession number AEVU00000000.

### Annotation

To maximize gene prediction accuracy, the gene structures of *Cordyceps *were predicted with a combination of different algorithms plus manual inspections [11,57]. The inconsistent open reading frames were individually subject to Blast searches against the NCBI curated refseq_protein database. The prediction with the best hit was selected. Pseudogene identification was conducted with the pipeline of PseudoPipe with default settings [58]. The potential secreted proteins of *C. militaris *and other fungal species included for comparison were predicted by SignaIP 3.0 analysis using a hidden Markov model [59]. Genome repetitive elements were analyzed by Blast against the RepeatMasker library (Open 3.2.9) [60] and with the Tandem Repeats Finder [61]. The transposases/retrotransposases were classified by Blastp analysis against the Repbase [62] plus manual inspections.

### Blast score ratio test

BSR tests [14] were conducted to compare the differences between *C. militaris *and the sequenced *Metarhizum *genomes and the plant pathogen *F. graminearum*, respectively. The BSR index for each reference protein is calculated by dividing the query bit score by the reference score and normalized from 0 to 1. A score of 1 indicates a perfect match while a score of 0 indicates no Blast match of a query protein in the reference proteome. The normalized pairs of BSR indices were then plotted using the Matlab (v7.0) program (Natick, Massachusetts, USA). The same analysis was conducted for the three genomes of *C. militaris*, *M. anisopliae *and *F. graminearum*.

### Orthology and phylogenomic analysis

In total, 2,106 orthologous proteins were acquired by a reciprocal Blast method with a cutoff E value of 1e-20 and a Blast alignment length greater than 60% of the query sequence. Corresponding orthologous gene protein sequences were aligned with Clustal X 2.0 and the concatenated amino acid sequences were used for the generation of a maximum likelihood phylogenomic tree with the program TREE-PUZZLE [63] using a Dayhoff model. The divergence time between species was estimated with the program r8s using a Langley-Fitch model [64] by calibration with the origin of the Ascomycota at 500 to 650 MYA [65].

### Protein family classifications

Whole genome protein families were classified by InterproScan [66] and Pfam [67] analysis. The families of proteases were identified by Blastp searching against the MEROPS peptidase database release 9.4 with a cutoff E value of 1e-20 [68]. The CYPs were named according to the classifications collected at the P450 database [69]. Transporters were classified based on the Transport Classification Database [70]. Kinases were classified by Blastp analysis against the KinBase database with a cutoff E value of 1e-10 [68]. Carbohydrate-active enzymes were classified by local Blastp searching against a library of catalytic and carbohydrate-binding module enzymes [68]. G-protein-coupled receptors were selected from the best hits to GPCRDB sequences [71] and by confirmation that they contained seven transmembrane helices with the amino terminus outside and the carboxyl terminus inside the plasma membrane. Homologs of the *Magnaporthe *Pth11-like GPCRs [30] were identified by local Blastp analysis with a cutoff E value of 1e-10. Putative *Cordyceps *virulence factors were identified by searching against the pathogen-host interaction database [72] with a cutoff E value of 1e-5, plus additional searches of known virulence genes reported in entomopathogenic fungi. Two sample *t-*tests were conducted to compare the differences in protein family sizes between insect and plant pathogens. Estimation of FDR of *P*-values was conducted using the program mafdr (Matlab 7.8.0.347(R2009a)).

### Analysis of genes involved in purine synthesis and secondary metabolism

To model the biosynthesis of cordycepin, the purine metabolic pathway in *C. militaris *was constructed based on the KEGG (Kyoto Encyclopedia of Genes and Genomes) annotations [73]. To identify NRPS, PKS or NRPS-PKS hybrid genes and gene clusters, the whole genome data set was subjected to analysis with the program SMURF with default settings [74]. Modulation analysis and domain extraction of different NRPS or PKS proteins were conducted by Blast searching against the SBSPKS database [75]. For phylogenetic analysis, the domain sequences were aligned with Clustal X 2.0 and the tree was generated using a Poisson model with 1,000 bootstrap replications and pair-wise deletions for gaps or missing data. The mycotoxin-encoding PKSs used in the analysis include *Gibberella zeae *PKS4 (ABB90283) and PKS13 (ABB90282) for the biosynthesis of zearalenones [76], *Aspergillus ochraceus *PKS (AAT92023) for ochratoxin [77], *A. clavatus *PatK (ACLA_093660) for patulin [78], *Gibberella moniliformis *Fum1p (AAD43562) for fumonisin [79], *Monascus purpureus *PKS (BAD44749) for citrinin [80], *Aspergillus flavus *PksA (AAS90093) for aflatoxin [81] and *A. nidulans *StcA (Q12397) for sterigmatocystin [82]. The mycotoxin-encoding NRPSs included in the analysis are *A. fumigatus *Glip (AAW03307) for gliotoxin [83], *Fusarium equiseti *NRPS (CAA79245) for enniatin [84], *Cochliobolus carbonum *NRPS (AAA33023) for HC-toxin [85] and *Tolypocladium inflatum *NRPS (CAA82227) for cyclosporin [86].

### Transcriptome analysis

Conidia of *C. militaris *from day 14 potato dextrose agar were inoculated into SDB and the undifferentiated mycelia harvested after a 72-hour incubation at 25°C, 180 rpm. The transcriptome of the mycelia provided a control for comparison with the transcriptomes of fruiting bodies. Chinese Tussah silkmoth (*Antheraea pernyi*) pupae were injected with 50 μl of a conidial suspension (5 × 10^6 ^conidia/ml) and incubated at 22°C in a 12:12 hour light:dark cycle for up to 14 days to allow emergence of fruiting bodies (a stage designated as FB1), 29 days for half-grown fruiting bodies (FB2) and 59 days, by which time fruiting bodies were mature (FB3) [11,12]. RNA was extracted with a Qiagen RNeasy kit plus on-column treatment with RNase-free DNase I (Germantown, Maryland, USA). Messenger RNA was purified and after reverse transcription into cDNA, the libraries were constructed for tag preparation according to the massively parallel signature sequencing protocol [87]. The tags were sequenced with an Illumina technique. We omitted tags from further analysis if only one copy was detected or it could be mapped to a different transcript. Other tags were mapped to the genome or annotated genes if they possessed no more than one nucleotide mismatch [11,88]. The abundance of each tag was converted to the value of transcripts per million (TPM) for each mapped gene for expressional comparison between samples. The significance of differential gene expression between samples and the FDR of *P*-values were estimated for each individual gene with a cutoff of *P *≤ 0.05 and FDR ≤0.001 [11,89]. The RNA_seq expression dataset is available at the NCBI's Gene Expression Omnibus under the accession code GSE28001.

## Abbreviations

ABC: ATP-binding cassette; bp: base pair; BSR: Blast score ratio; CCM: *Cordyceps militaris*; CYP: cytochrome P450; DMAT: dimethylallyl tryptophan synthase; FDR: false discovery rate; GH: glycoside hydrolase; GI: genomic island; GPCR: G-protein coupled receptor; MAA: *Metarhizium anisopliae*; MAC: *Metarhizium acridum*; MAPK: mitogen-activated protein kinase; MAT: matting-type; MFS: major facilitator superfamily; MYA: million years ago; NRPS: non-ribosomal peptide synthetase; PKA: protein kinase A; PKS: polyketide synthase; RIP: repeat-induced point mutation; RNR: ribonucleotide trisphosphate reductase; SDB: Sabouraud dextrose broth; TF: transcription factor; TPM: transcripts per million tags.

## Authors' contributions

CSW initiated and designed the study. PZ, YLX, GHX, CHX, YH and YZ annotated genes and performed protein family analysis; GHX and HX performed phylogenetic and transcriptome analysis; PZ and SWZ conducted fruiting body induction, and DNA and RNA extraction; HJZ, SYW and GPZ performed genome sequencing and assembly; PZ and GHX performed transcriptome analysis; CSW and RJSL wrote the paper. All authors read and approved the final manuscript.

## Supplementary Material

Additional file 1 **Comparative genomics analysis of *C. militaris***. The file contains additional information on genomic properties and comparative gene family analysis of *C. militaris *with other fungi comprising 19 tables provided in separate excel sheets. Table S1 summarizes major protein family sizes of different fungal species. Table S2 provides a comparison of transposase genes among three insect pathogens. Table S3 lists the pseudogenes present in the genomes of three insect pathogens. Table S4 summarizes the protein families putatively involved in pathogen-host interactions. Table S5 compares the proteases in different fungal genomes. Table S6 lists the serine and aspartyl proteases in three insect pathogens. Table S7 lists the glycoside hydrolase families in different fungal genomes. Table S8 compares the cytochrome P450 genes in three insect pathogens. Table S9 summarizes the membrane transporters in different fungal genomes. Table S10 compares the G-protein-coupled receptors in three insect pathogens. Table S11 lists the protein kinases in three insect pathogens. Table S12 provides the information of mating- and sexuality-related genes. Table S13 lists the genes putatively involved in purine metabolisms in three insect pathogens. Table S14 summarizes the presence/absence of patulin biosynthesis homologous genes in *C. militaris*. Table S15 summarizes the presence/absence of T-2 toxin biosynthesis homologous genes in *C. militaris*. Table S16 summarizes the information from RNAseq analysis. Table S17 lists the 100 most highly expressed genes in *C. militaris *at different growth stages. Table S18 lists the transcriptional data of sexuality- and fruiting-related genes. Table S19 compares the expression data of genes putatively involved in signaling and transcription controls. Click here for file

Additional file 2 **Figures that provide support information for the main text**. Figure S1 provides support for RIP occurring in *C. militaris*. Figure S2 provides support for the lack of the pentose metabolic pathway in *C. militaris*. Figure S3 provides a phylogeny analysis of fungal ribonucleotide reductases. Figure S4 provides the phylogeny and modular analysis of *C. militaris *NRPSs. Figure S5 provides a phylogeny analysis of fungal dimethylallyl tryptophan synthases. Figure S6 provides the gene transcription profiles between different samples. Click here for file
