# Supplementary material for: Genome sequence of the insect pathogenic fungus Cordyceps militaris, a valued traditional chinese medicine
Source: Genome Biol. 2011 Nov 23;12(11):R116. doi: 10.1186/gb-2011-12-11-r116 (PMC3334602; doi:10.1186/gb-2011-12-11-r116)
Supplement: Additional file 2 — Figures that provide support information for the main text. Figure S1 provides support for RIP occurring in C. militaris. Figure S2 provides support for the lack of the pentose metabolic pathway in C. militaris. Figure S3 provides a phylogeny analysis of fungal ribonucleotide reductases. Figure S4 provides the phylogeny and modular analysis of C. militaris NRPSs. Figure S5 provides a phylogeny analysis of fungal dimethylallyl tryptophan synthases. Figure S6 provides the gene transcription profiles between different samples. [file gb-2011-12-11-r116-S2.DOCX]

**Additional file 2**

**
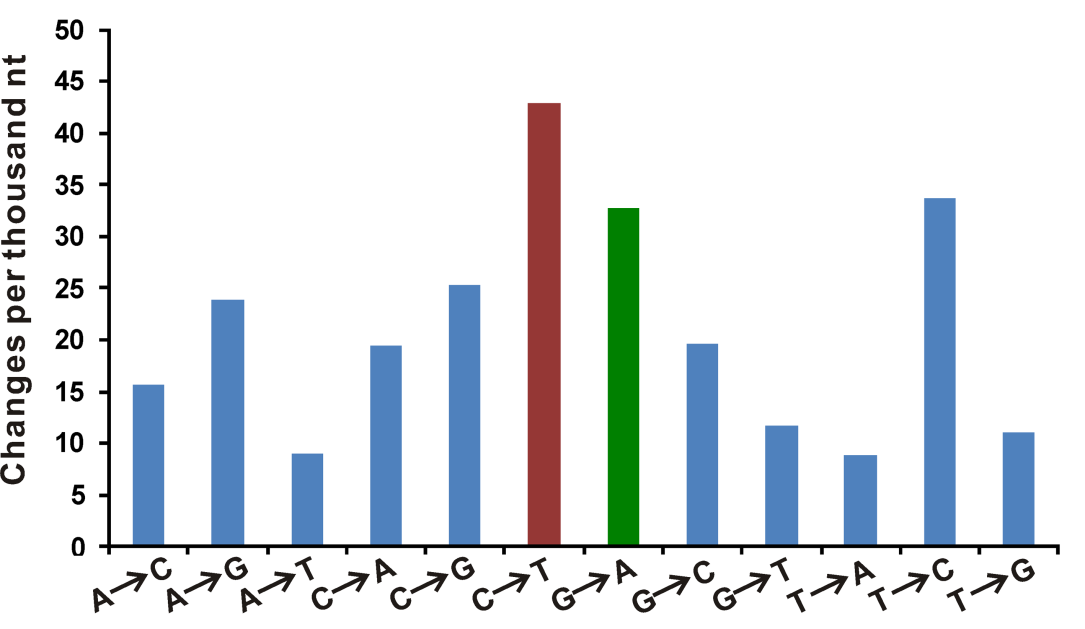
**

**Figure S1 Estimation of nucleotide mutation rates for the *C. militaris* paralogous genes** **showing >70% identity in paired protein sequences.** For calculations, the gene of each pair with the greater similarity to the ortholog from *F. graminearum* was used as the reference.

**
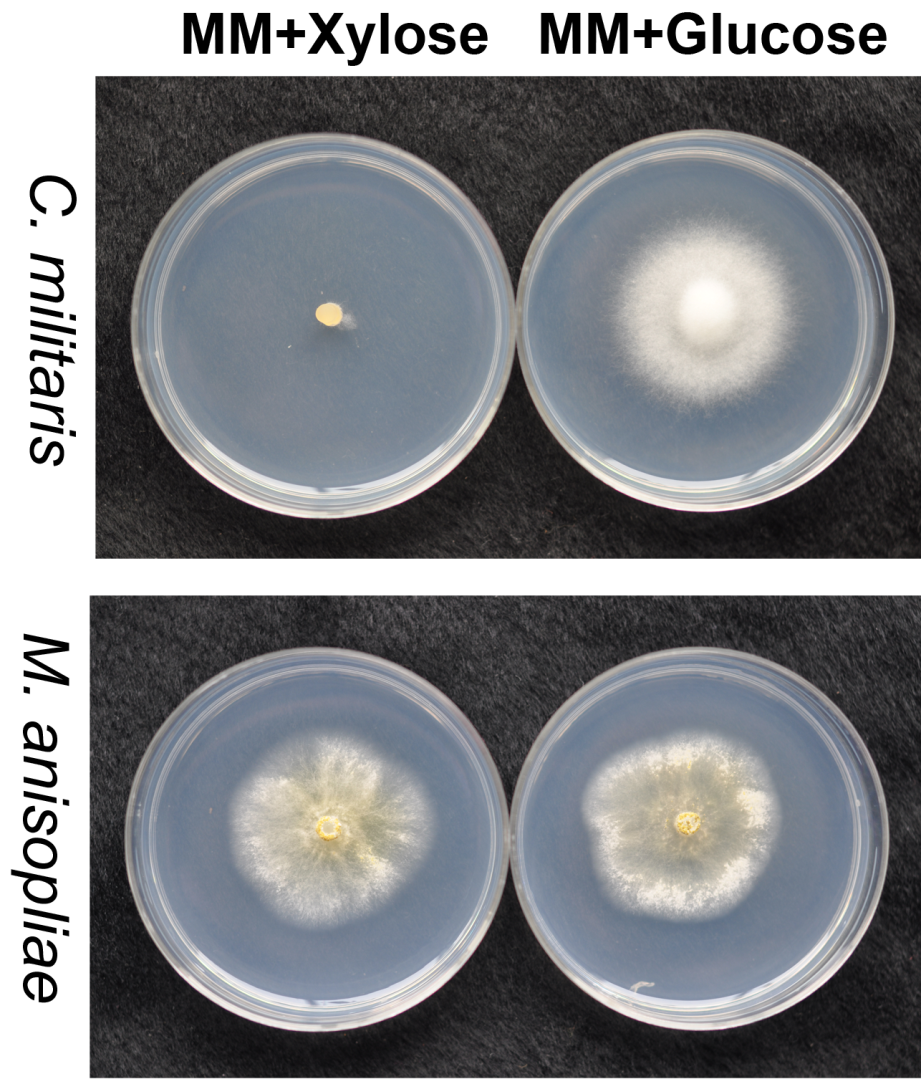
**

**Figure S2. Growth test on xylose medium.** The minimal medium (NaNO_3_ 6 g/l, KCl 0.52 g/l, MgSO_4_.7H_2_O 0.52 g/l, KH_2_PO_4_ 0.25 g/l) was supplemented with either 1% (10 g/l) xylose or glucose. The fungi were inoculated and incubated for 12 days. In contrast to *M. anisopliae*, *C. militaris* failed to grow on xylose medium.

**
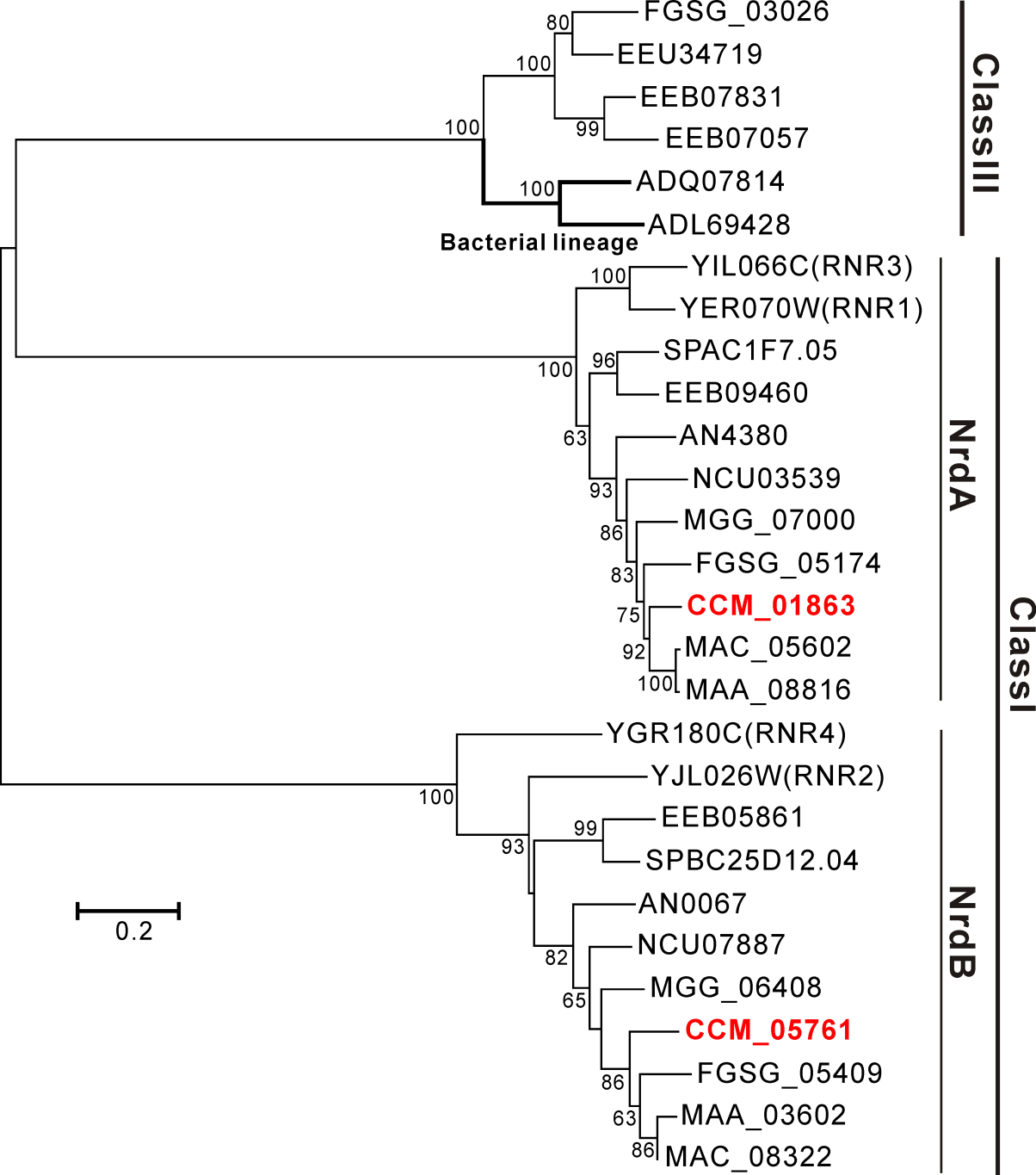
**

**Figure S3 Phylogenic analysis of selected fungal ribonucleotide reductases (RNRs).** The neighbor-joining tree was generated using protein sequences with 1000 bootstrap replicates under a Poisson substitution model. Most fungi, including *C. militaris*, only have NrdA and NrdB types of class I ribonucleoside diphosphate reductases but the plant pathogens *F. graminearum* (FGSG_03026) and *Nectria haematococca* (EEU34719) have one copy each, while *Schizosaccharomyces japonicas* (EEB07831 and EEB07075) has two copies of bacterial-like class III anaerobic ribonucleotide triphosphate reductases. The sequences from *C. militaris* are highlighted in red.

**
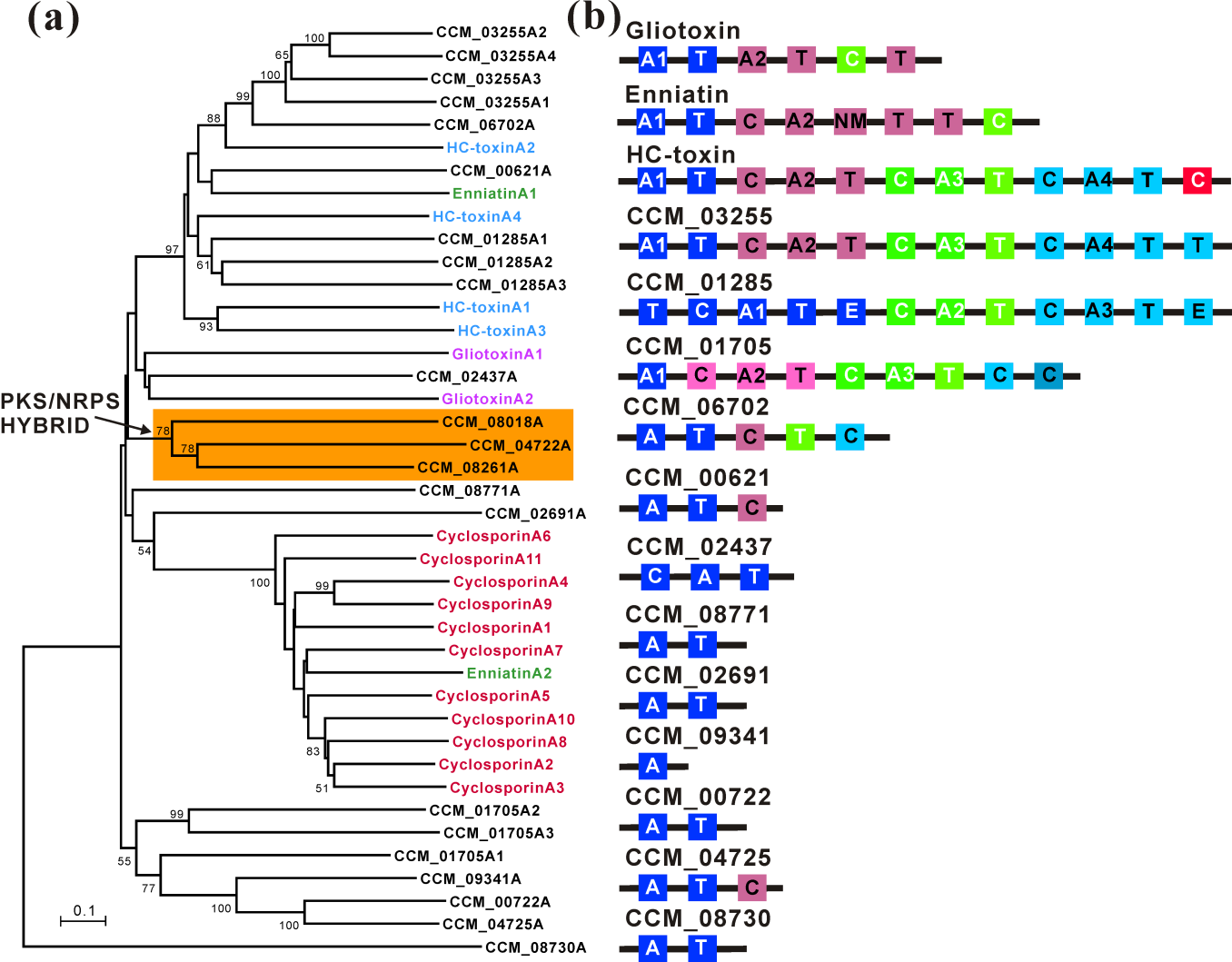
**

**Figure S4 Phylogenetic and modular analysis of *C. militaris* non-ribosomal peptide synthetases (NRPS) compared with those involved in production of known mycotoxins.** (**a**) Neighbor-joining tree showing the relationships of adenylation (A) domain sequences. (**b**) Modulation and comparison of *C. militaris* NRPSs with known mycotoxin synthetases. The PKS-NRPS hybrid proteins CCM_04722, CCM_08261 and CCM_08018 are not included in this analysis. The mycotoxin related NRPSs include those for the biosynthesis of gliotoxin (*A. fumigates*, AAW03307), enniatin (*F. equiseti*, CAA79245) and HC-toxin (*Cochliobolus carbonum*, AAA33023). The cyclosporin produced by the *Tolypocladium inflatum* NRPS (CAA82227) is a commercial drug. The definitions of domains are T for protein domain involved in thiolation; C for condensation; NM for N-methylation and E for epimerization.


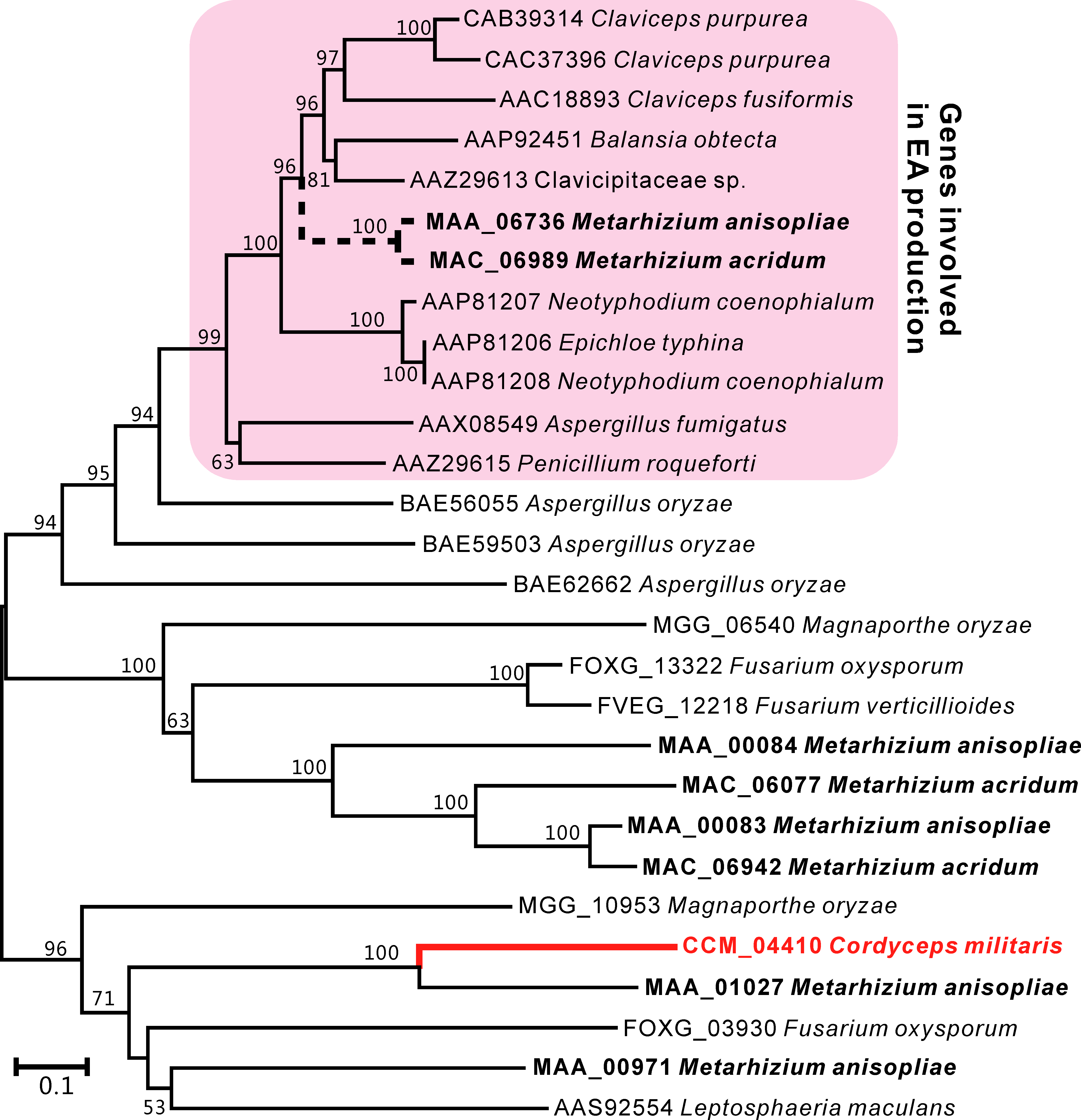


**Figure S5 Phylogenetic analysis showing the evolutionary relationships of fungal dimethylallyl tryptophan synthases (DMATs).** The neighbor-joining tree was generated using protein sequences under a Poisson model with 1,000 bootstrap replications and a pairwise deletion for gaps or missing data. Except for MAA_06736 from *M. anisopliae* and MAC_06989 from *M. acridum*, the DMAT sequences in the shadowed clade including *Claviceps* spp. are experimentally confirmed to be involved in the production of ergot alkaloids (EA). The putative DMAT CCM_04410 from *C. militaris* (red) is outside the EA clade, i.e. not responsible for EA production.


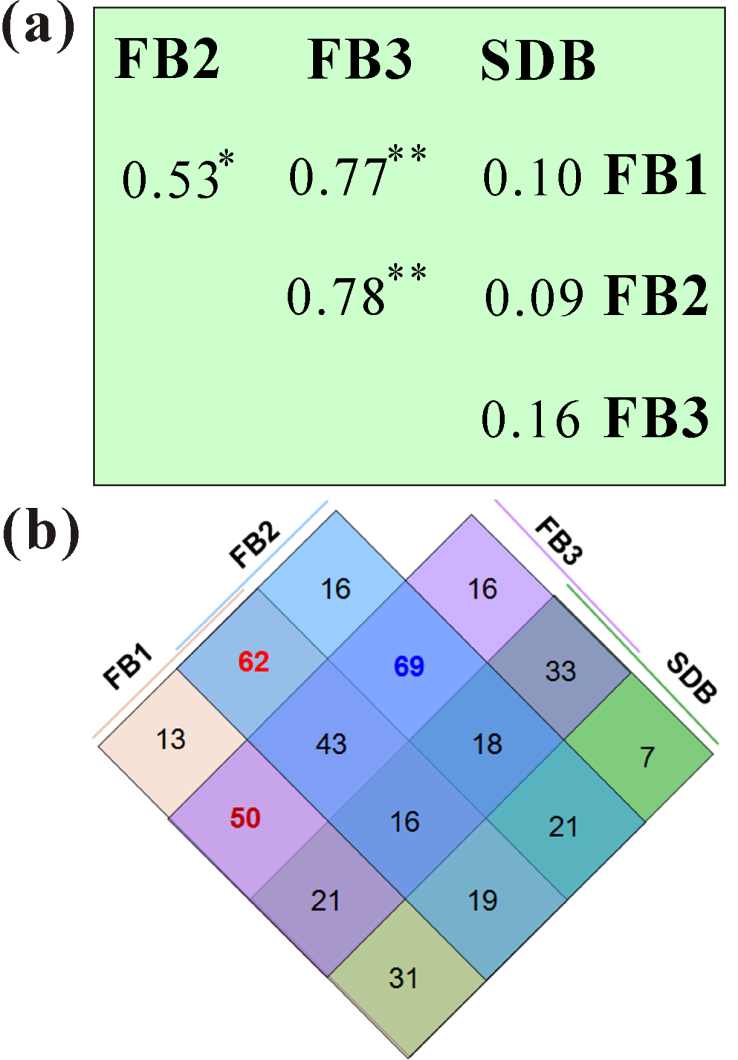


**Figure S6 Gene expression profiling. (a)** Pearson correlation analysis of gene expression data between different samples. * means a significance level at *P* ≤ 0.05; **, *P* ≤ 0.01. (**b**) Venn diagram analysis of 100 most highly expressed genes between samples.
